# A machine-compiled database of genome-wide association studies

**DOI:** 10.1038/s41467-019-11026-x

**Published:** 2019-07-26

**Authors:** Volodymyr Kuleshov, Jialin Ding, Christopher Vo, Braden Hancock, Alexander Ratner, Yang Li, Christopher Ré, Serafim Batzoglou, Michael Snyder

**Affiliations:** 1 Department of Computer Science, Stanford University, Stanford, CA 94305 USA; 2 Department of Genetics, Stanford University School of Medicine, Stanford, CA 94305 USA; 3 Department of Medicine, University of Chicago, Chicago, IL 60637 USA

**Keywords:** Data mining, Data publication and archiving, Genetic databases, Genome-wide association studies

## Abstract

Tens of thousands of genotype-phenotype associations have been discovered to date, yet not all of them are easily accessible to scientists. Here, we describe GWASkb, a machine-compiled knowledge base of genetic associations collected from the scientific literature using automated information extraction algorithms. Our information extraction system helps curators by automatically collecting over 6,000 associations from open-access publications with an estimated recall of 60–80% and with an estimated precision of 78–94% (measured relative to existing manually curated knowledge bases). This system represents a fully automated GWAS curation effort and is made possible by a paradigm for constructing machine learning systems called data programming. Our work represents a step towards making the curation of scientific literature more efficient using automated systems.

## Abstract (web summary)

Most databases of genotype-phenotype associations are manually curated. Here, Kuleshov *et al*. describe a machine curation system that extracts such relationships from the GWAS literature and synthesizes them into a structured knowledge base called GWASkb that can complement manually curated databases.

## Introduction

Genome-wide association studies (GWAS) are widely used for measuring the effects of genetic variants on human traits^1^. About 2500–3000 studies have been performed to date^2,3^; their results are used to estimate disease risks^4,5^, to understand the function of specific genomic regions^6,7^, and to train algorithms that predict the effects of new variants.^8^

Most applications require the GWAS associations to be accessible in a structured format amenable to automated analysis by a computer. Several manual curation efforts are underway to catalogue published GWAS associations into structured databases^2,3^; however, these efforts require time, domain expertise, and can be prone to errors. As more studies are published, the cost of curation is expected to increase.

Here, we describe GWASkb, a machine-compiled knowledge base of thousands of genotype–phenotype associations. It represents a fully automated GWAS machine curation effort, made possible by a paradigm for constructing machine learning systems called data programming.^9,10^ GWASkb is constructed from 589 open-access GWAS publications, and recovers > 6000 associations from these publications at an estimated recall of 60–80% and with an estimated precision of 78–94% (both depending on stringency criteria and measured relative to existing manually curated knowledge bases over the same input dataset).

GWASkb is useful to curators as it provides a large dataset of candidate associations for inclusion into existing knowledge bases. These associations are also useful to scientists and clinicians in order to study the genetic basis of human traits and to estimate the disease risks of individuals. To facilitate these use cases, we are making available the code used to create GWASkb, and we also provide an online tool for browsing the associations found by the system. More broadly, our work may form the basis for further efforts to curate Mendelian variants and other biological information.

## Results

### Automating biomedical literature curation with GWASkb

**Fig. 1:**
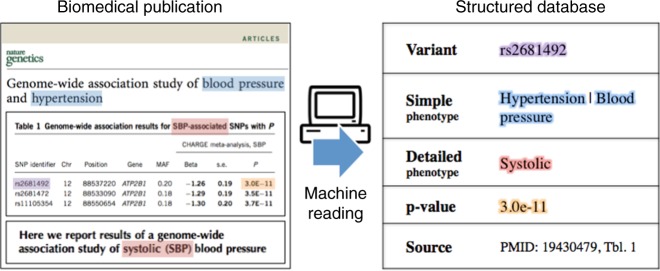
The automated information extraction system used to compile GWASkb. The GWASkb system takes as input a set of biomedical publications retrieved from PubMed Central (left) and automatically creates a structured database of GWAS associations described in these publications (right). For each association, the system identifies a genetic variant (purple), a high-level phenotype (pertaining to all variants in the publication), a detailed low-level phenotype (specific to individual variants, if available; red), and a *p* value (orange). Acronyms are also resolved (red)

At a high level, we extract genotype–phenotype relations from the biomedical literature and place them in a structured database (Fig. 1). A typical association consists of a genetic variant, its associated phenotype, and a p-value indicating the significance of the association (see Supplementary Note 3). GWASkb collects these three specific characteristics. Associations also possess additional properties that our system does not yet process; these include an effect size, a risk allele, a target population, and others. Finally, we support our findings with evidence from publications (identified by their Pubmed ID), which are locations inside the document.

When reporting phenotypes, human-curated databases can be at times very specific (e.g., high systolic blood pressure) and at other times less so (e.g., heart disease). In GWASkb, we report simple and precise phenotypes; the former is a high-level description that applies to every variant in the paper (e.g., effects of proteins on inflammation), while the latter is a detailed description that, when available, applies to specific variants (e.g., the name of a specific protein).

We also aim to collect a large set of associations that can be refined by users according to their needs. This approach offers more flexibility than collecting only high-confidence relations, a common approach for manual curation efforts.

### Creating GWASkb using IE algorithms

We have structured the system used to generate GWASkb into a set of five components that extract three key pieces of information: genetic variants, their phenotypes, and their *p* values.

The first component of our system parses the title and abstract of every paper to identify a simple phenotype that will be associated with all its variants. The second component parses the body of the paper to find tuples of Reference SNP cluster IDs (RSIDs) and their associated precise phenotypes. Often, the precise phenotype is abbreviated (e.g., body mass index (BMI)) and a third component attempts to resolve these abbreviations (e.g., BMI). A fourth component extracts *p* values in the form of (rsid, *p* value) tuples. Finally, the fifth component constructs a single structured database from all these results.

**Fig. 2:**
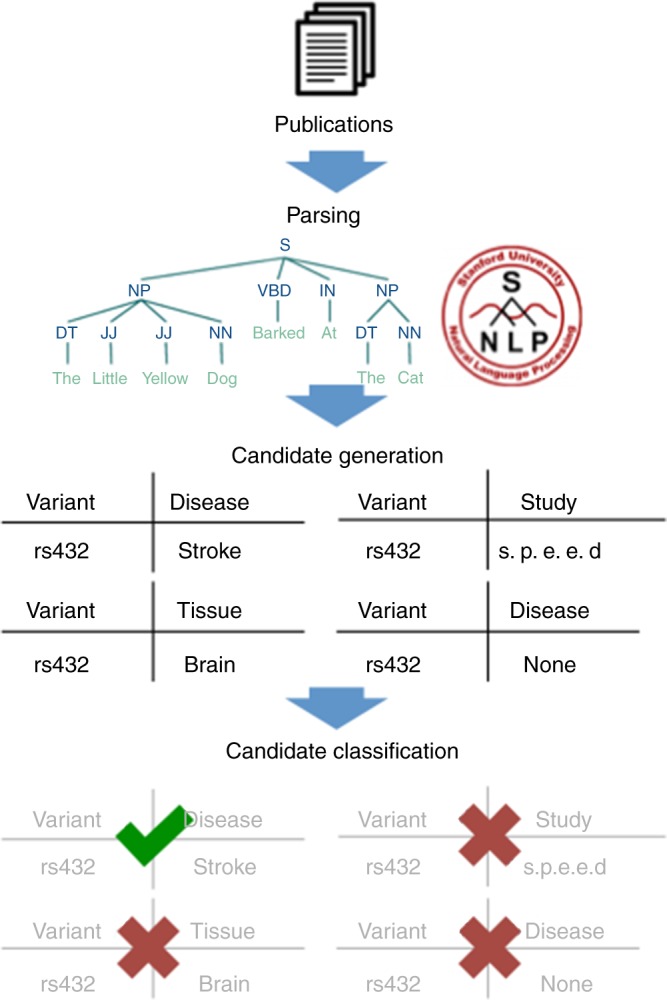
General structure of a GWASkb system module. The system contains separate modules for extracting variants, phenotypes, *p* values, and for resolving acronyms. Each module consists of three stages. At the parsing stage, we process papers using the Stanford CoreNLP pipeline, performing full syntactic parsing. Next, given a target relation (e.g., variant-phenotype), we generate a large set of candidates, some of which could be correct instances of the target object on relation. Then, at the classification stage, we determine which candidates are correct using a machine learning classifier

The components of our information extraction (IE) system are composed of three stages: parsing, candidate generation, and classification (Fig. 2). Parsing is performed with Snorkel^10^, a knowledge base construction framework for documents with richly formatted data (data expressed via textual, structural, tabular, and/or visual cues), such as XML documents. Content is first parsed for structure—the XML tree is traversed and converted into a hierarchical data model with text assigned to tables, cells, paragraphs, sentences, etc. Then, each sentence or cell is parsed for content using the Stanford CoreNLP pipeline^11^, which performs sentence tokenization, part-of-speech tagging, and syntactic parsing. In candidate generation, we identify in the text mentions of some target relation (e.g., *p* value/rsid pairs). Regular expressions or dictionaries are used to identify candidates that may be valid instances of the relation we are looking for (erring on the side of high recall over high precision). Finally, in the classification stage, we determine which of these candidates are actually correct relation mentions using a machine learning classifier. We use a Naive Bayes classifier with a small number of hand-crafted features (between 4 and 12), and we train the model using the recently proposed data-programming paradigm.^9^

One of the most significant bottlenecks in developing machine learning-based IE systems is collecting large sets of hand-labeled training data. Data programming is a paradigm for training models using higher-level, less precise supervision to avoid this bottleneck.^9^ In this approach, users write a set of LFs: black-box functions that label data points, and that can subsume a wide variety of heuristic approaches such as distant supervision^12^—where an external knowledge base is used to label data points—regular expression patterns, heuristic rules, and more. These LFs are assumed to be better than random, but otherwise may have arbitrary accuracies, may overlap, and may conflict. A generative model is used to learn their accuracies and correlations from unlabeled data. The predictions of this model can then be used for classification, or to generate labels for a second, discriminative model. For further details see Supplementary Note 1.

In this work, we use data programming to train a generative Naive Bayes classifier over a small number (4–12) of hand-crafted LFs (Supplementary Note 6). We then directly apply these probabilistic labels as predictions. We refer the reader to the appendix for more details.

### Reproducibility

In order to make our results fully reproducible, we have released Jupyter notebooks that can be used to generate GWASkb and recreate most of our figures and tables. The notebooks and the source code used to generate GWASkb is freely available on GitHub at github.com/kuleshov/gwaskb.

In addition, we have built an interactive website (see Supplementary Note 10) that enables users to browse associations that have been extracted in GWASkb. Users can search the data by study, phenotype or variant rsid. The entire dataset can also be downloaded from GitHub in CSV format or using the link provided in Supplementary Note 11.

### Machine reading helps automate GWAS curation

We compiled GWASkb from 589 open-access GWAS papers, which are papers that are not affected by copyright restrictions that limit our right to perform automated text mining. These papers represent approximately 25% of studies recorded at the time of writing in the NHGRI-EBI GWAS Catalog, a popular human-curated database. We retrieved these papers from the PubMed Central (PMC) repository in XML format and passed the XML source code as input to the IE system. Note that our system can also be deployed on nonopen-access papers if a user has legal rights to do so.

Genome-wide associations are typically identified in a discovery cohort and then replicated in a separate replication cohort. Some curation projects (such as GWAS Catalog) only include associations that have been successfully replicated, while others (such GWAS Central) tend to include most associations. GWASkb follows the latter approach; this offers more flexibility and allows researchers to refine the data according to the level of confidence that best suits their needs.

For the purpose of evaluating the precision and recall of our system, we formed a dataset of all automatically extracted associations that were determined to be significant at *p* < 10^−5^ in at least one experiment in the study (such as in one cohort or one statistical model). This criterion recovered a significant number of associations present in existing databases, while maintaining sufficiently high precision (Table 1).

**Table 1:** Numbers of associations contained in different GWAS databases; statistics are over open-access papers

Database	Papers	Associations	Unique associations
GWAS Catalog	589	8384	>2026
GWAS Central	516	5914	>364
GWASkb (ours)	589	6231	>2777

Unique associations are contained in one database and in none of the others. Human curated databases (GWAS Catalog and GWAS Central) significantly differ in their scope. Our machine-compiled repository (GWASkb) automatically recovers a large fraction of known results and also finds a comparable number of unique associations

It is important to note that our inclusion criterion is different from the one used by databases such as the GWAS Catalog, which typically includes associations that are significant in a combined discovery and replication cohort, unless only discovery data are available and no replication was attempted. Our criterion approach of accepting all associations with their metadata is more flexible, as it allows researchers to refine the data according to their needs. A disadvantage of this approach is that it is also includes low-confidence associations, such as ones that have not been replicated, that originate from an earlier study, or that may arise from non-GWAS experiments. A lower-confidence dataset may still be useful for certain applications, such as for testing whether certain pathways are enriched for associated variants. However, this also requires manually filtering variants that do not meet the significance threshold for other applications, which can be burdensome. To assist with this process, we are releasing metadata that helps identify the cohort associated with a given variant (see Supplementary Note 9). This metadata can later be used to train classifiers that automatically identify the target cohort.

### GWASkb recovers up to 80% of manually curated associations

GWAS Central and GWAS Catalog contain, respectively 3008 and 4023 accessible associations linked to the 589 open-access studies. These associations are defined as tuples of PubMed ID, variant RSID, phenotype, and *p* value for which the RSID is contained in the open-access XML content made available through PubMed Central. For GWAS Catalog we use the reported trait for our analysis rather than the ontology terms (EFO). To measure recall relative to human-curated databases, we need to determine whether each (PubMed ID, RSID, phenotype, *p* value) tuple reported in GWASkb is also present in the human-curated database. This requires deciding whether phenotypes reported in GWASkb are equivalent to ones reported by human curators; to determine this, we manually created a mapping between GWASkb phenotypes and phenotypes reported in either GWAS Central or GWAS Catalog for the same PubMed ID and RSID (see Methods).

Since databases use different levels of precision to describe traits (e.g., smoking behaviors vs. cigarette packs per day), we also specify whether our reported phenotype is exact or approximate; in the latter case, it is still useful, but lacks some detail. Table 2 contains examples of relations contained in GWASkb at different levels of precision.

**Table 2:** Examples of associations identified by GWASkb

Study	Association	Simple phenotype (GWASkb)	Precise phenotype (GWASKb)	*p* Value (GWASkb)	Phenotype (GWAS Cat)	*p* Value (GWAS Cat)
Genome-wide pharmacogenomic study of metabolic side effects to antipsychotic drugs	*rs17661538*	Antipsychotic drugs/metabolic side effects	Clozapine—Triglycerides	1.00E−06	Clozapine-induced change in triglycerides	1.00E−06
Genome-wide meta-analysis identifies seven loci associated with platelet aggregation in response to agonists	*rs12566888*	Platelet aggregation	–	5.00E−19	Platelet aggregation, and epinephrine	5.00E−19
A genome-wide association study of the Protein C anticoagulant pathway	*rs13130255*	Protein C	funcPS	3.00E−06	Anticoagulant levels (funcPS)	3.00E−06

Associations can be classified as correct (*rs17661538*), partially correct (*rs12566888*; the precise phenotype is missing) and incorrect (*rs13130255*). We also compare these associations to their corresponding entries in the GWAS Catalog.

The dataset reported in GWASkb contained 2487 (82%) associations from GWAS Central with approximately correct phenotypes, as well as 3245 (81%) associations from the GWAS Catalog. It also recovered 1890 (63%) associations from GWAS Central and 2762 (69%) associations from GWAS Catalog with full accuracy on the phenotype. Some associations were not correctly recovered because the GWASkb reported phenotype was incorrect: 89 (3%) for GWAS Central and 147 (4%) for GWAS Catalog. In the remaining cases, we were not able to report the variant itself. The main causes of this are when the variants are expressed only in the text and not in tables, or when the format of the table is particularly difficult to parse (e.g., when multiple RSIDs and *p* values are reported in the same row). Overall, GWASkb recovered 81–82% of manually curated associations at a level of quality that will be useful in many applications.

### Machine curation uncovers useful new associations

In total, GWASkb contains 6422 associations within the 589 input papers, 2959 (46%) of which could not be mapped to GWAS Catalog or GWAS Central. We investigated this further by first manually inspecting a random subset of 100 novel associations (with independent validation from two independent annotators). We found that 88 associations fully met the specifications of our system, 7 were incorrect, and 5 were originally identified by a different study (and referenced as background material). Most of the errors of our system can be attributed to incorrect phenotypes.

Of the 88 associations matching system specifications, 44 were not significant at 10^−5^ in all cohorts, hence were excluded from GWAS Catalog for scientific reasons. We report these variants because they may still be useful in applications in which a noisy dataset is acceptable. Another 36 were excluded because they were in the same locus as a more significant variant; however, these were generally not in perfect linkage disequilibrium (LD) and 27 were in weak LD with the GWAS Catalog variant (*r*^2^ < 0.5 as determined by the LDLink tool). We argue for cataloguing these variants, as the LD cutoff for what constitutes a significant variant may depend on the scientific application. Another eight variants were not included because they were determined to be significant in both the target study and an earlier study (note that GWAS Catalog guidelines state that such variants should be catalogued).

### LD between new and existing variants

To validate the novel variants found by our system, we conducted a series of analyses aimed at characterizing the variants’ function.

**Fig. 3:**
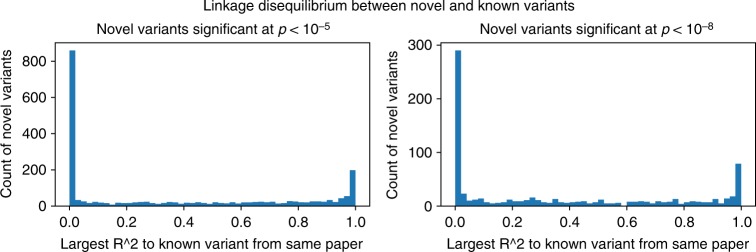
Linkage disequilibrium between GWASkb variants not present in existing human curated databases and variants from the GWAS Catalog. We use the 1000 Genomes dataset to estimate the *r*^2^ metric between pairs of variants, and report distances from each GWASkb variant to the most correlated GWAS Catalog SNP reported in the same paper. The distribution of *r*^2^ scores is highly multimodal; many GWASkb variants are uncorrelated (*r*^2^ = 0) with GWAS Catalog SNPs. Reported *p* values are generated from *χ*^2^ test

First, we reasoned that detected variants may be in LD with known variants (because they originate from the same LD block), or among themselves, thereby inflating our number of truly novel associations. We estimated LD from the Thousand Genomes dataset (see Supplementary Note 2); Fig. 3 shows the histogram of *r*^2^ distances between each novel variant, and its closest variant in the GWAS Catalog. The distribution of *r*^2^ scores is highly multimodal, with large peaks at *r*^2^ = 1, and many more at *r*^2^ = 0.

Using a threshold of *r*^2^ > 0.5, we filtered our set of new [pmid, rsid, phen, pvalue] associations from 3170 to 1494 by removing variants in LD with known manually curated variants; of the 1676 variants that we eliminated, 765 were not in the 1000 Genomes database or their closest previously known variant was not in the database; the remaining 911 single-nucleotide polymorphisms (SNPs) were in LD with known variants. We further reduced this set to 1304 associations by eliminating novel variants that were in LD with each other. Thus, although many variants are in LD with known variants, over 40% of our discovered variants do not appear to be linked to variants previously identified in GWAS databases.

Although our system reported multiple variants from the same LD block, these variants may still be useful, since we do not know which variant an LD block is truly causal and the *r*^2^ cutoff for defining LD blocks is somewhat arbitrary and may vary. We believe that filtering should be performed by the user, depending on their goal; this is also the approach taken by the GWAS Central repository.

### Comparison to other approaches for estimating significance

Our second analysis focuses on the biological function of the novel variants. We focus on two large classes of phenotypes: neurodegenerative diseases (ND; 27 traits, including Autism, Alzheimer’s, Parkinson’s, etc.) and autoimmune disorders (AI; 23 traits, including Diabetes, Arthritis, Lupus, etc.); for the analyses below, we consider the subset of variants that are not in LD with any variant in the GWAS Catalog or GWAS Central (283 ND SNPs and 155 AI SNPs).

We also collected two sets of genes that were found to be highly expressed in brain cells as well as in blood cells; specifically, we reasoned that SNPs associated to neuropsychiatric and autoimmune diseases should be more highly enriched near genes expressed in brain and immune cells, respectively. Indeed, we found that variants associated with ND diseases (32 ND SNPs in total) occurred significantly more often within 200 kbp of genes with preferential brain expression, while variants associated with AU traits (15 variants in total) were found more frequently near genes with preferential blood expression (*χ*^2^ test: *p* < 0.05; see Supplementary Note 2).

We should note, however, that the vast majority of ND and AU variants were found far from coding regions. To test whether this set of SNPs also make biological sense, we used GREAT^13^, a tool which annotates the function of variants in intergenic areas of the genome. In particular, GREAT links intergenic regions with Disease Ontology (DO) terms, and outputs terms that are significantly enriched for a particular set of variants. When we applied GREAT to ND SNPs, we found a strong enrichment in regions known to play a role in ND-related phenotypes, such as cognitive disease (*p* < 10^−32^), dementia (*p* < 10^−23^), and neurodegenerative disease (*p* < 10^−23^). Similarly, AI variants were significantly associated with AI-related terms, the most significant of which were disease by infectious agent (*p* < 10^−27^), viral infectious disease (*p* < 10^−19^), and autoimmune disease (*p* < 10^−17^). In fact, the top 20 DO terms for either set of variants were all exclusively associated with the correct family of phenotypes (Supplementary Tables 1 and 2). Hence, our predicted variants were consistent with external annotations.

### Examining the effect sizes of novel GWASkb variants

**Fig. 4:**
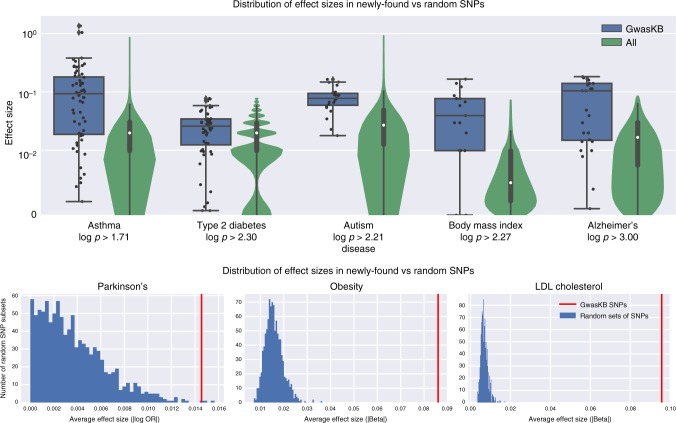
Visualizing the effect sizes of variants identified in GWASkb. *Top:* We compare the distribution of effect sizes (absolute values of beta coefficients or log odds ratios; data from LD Hub) of variants identified in GWASkb (blue) to that of all variants (green) for multiple traits. Blue variant effect sizes cluster away from zero and follow a different distribution (Kolmogorov–Smirnov test). In the boxplots, center lines represent medians, the box boundaries span the interquartile range, and the whiskers extend to the minimum and maximum observations excluding statistical outliers. *Bottom:* We subsample 1000 random sets of variants with the same number of elements as the set of GWASkb SNPs for a given disease; the average effect size of GWASkb variants (red) is higher than that of the random subsets (blue). In all settings, we only look at novel GWASkb variants not present in existing human-curated repositories

Finally, we analyzed the magnitude with which novel variants affect their predicted phenotypes and other, related traits. Specifically, we used freely available GWAS summary statistics from the LD Hub project^14^ to assess the distribution of SNP effect sizes across novel variants and compared them to those of random SNPs. We focused on the 11 most frequent traits in our dataset for which summary statistics were available; for each trait, we identified an LD Hub study that provides effect sizes (in the form of beta coefficients or log odds ratios) for that trait. Figure 4 compares the distribution of effect sizes of the novel variants identified in GWASkb to the distribution of effects sizes for all SNPs, again restricting to variants that show no LD with other variants in GWAS databases. Whereas the distribution of random SNPs is centered around zero, as one would expect, novel SNP effect sizes appear to follow a different distribution (Kolmogorov–Smirnov test; see Fig. 4 and Supplementary Figs. 1 and 2) and tend to have significantly higher magnitudes than expected.

## Discussion

Curation of the literature is critical because if GWAS associations are not recorded in a database, they are effectively missing for many practical purposes, such as for training machine learning systems to predict SNP function. GWAS studies are also costly (often involving genotyping tens of thousands of subjects), and it thus a waste of research funding to not fully record their results. Systems like the one used to create GWASkb can assist the curation process by providing useful candidates to human curators.

Most existing GWAS databases are constructed by human curators, who are expert scientists with advanced training that enables them to understand and parse complex study designs. Manual curation yields accurate and trusted results, albeit at a high labor cost (e.g., GWAS Catalog associations are verified by a second curator for maximum accuracy). An alternative to curation is to ask authors to directly report their findings online. This is already possible within GWAS Central, although in practice few authors choose to do this. In addition, past studies still need to be curated. An ideal solution appears to involve a combination of authors, machines, and curators.

However, manual-curation is a difficult task, and can miss certain associations. Curating papers is often a tedious task involving browsing through highly technical material in search of short snippets of text. Humans are generally not well-suited to this kind of work: they may accidentally skip table rows, or become tired and skip a paragraph. Curation also requires understanding advanced technical concepts such as LD or multiple hypothesis testing. This makes the task unsuitable for crowdsourcing approaches.

Computers, on the other hand, do not suffer from the aforementioned limitations: they excel at repetitive work and only need to be programmed by experts once. Crucially, even though machines make errors, these errors are systematic, not random: one may follow an iterative process of fixing these errors and redeploying the system, until a sufficient level of accuracy is reached. Redeploying our system takes on the order of hours, while asking humans to return and correct their errors would take at least months.

Of course, humans also have many advantages over machines. Indeed, the sets of GWASkb and human-curated associations were quite distinct. The most accurate and complete GWAS database is in fact a combination of both sources. In the future, we see curation as a collaboration between humans and machines.

Extracting structured relations from unstructured text is subject of the field of IE^15^. IE is widely used in diverse domains such as news^16^, finance^17^, geology^18^, and in the biomedical domain. In the biomedical setting, IE systems have been used to parse electronic medical records^19^, identify drug–drug interactions^20^, and associate genotypes with drug response^21^. A considerable amount of effort has gone into uncovering gene/disease associations from biomedical literature^22^. Our approach, however, takes a different approach, as it attempts to identify the effects of individual variants. Recently, Jain et al.^23^ applied IE to the GWAS domain; their work focused on creating extractors for two specific relations: paper phenotypes and subject ethnicities; these extractors achieved an 87% precision-at-2 and an 83% F1-score on the two tasks, respectively. In contrast, our works introduces an end-to-end system that extracts full (phenotype, rsid, *p* value) relations comparable to ones found in hand-curated databases.

Beyond GWAS studies, literature curation efforts are currently underway in cancer genomics, pharmacogenomics, and many other fields. Our findings hint at the possibility of using machine curation there as well.

The GWAS domain is in many ways easier than others since variants have standardized identifiers and a lot of information is structured in tables. Nonetheless, it allows us to demonstrate the importance of machine curation and to develop a core system that can be generalized to other domains. Within the GWAS setting, our system can be further improved by extracting additional information about variants (e.g., risk alleles and odds ratios). In addition, the current version of our database does not contain crucial study metadata such as study design, study stage, ancestry information, statistical methodology, etc. These are typically curated by human experts.

In summary, we have introduced a machine reading system for extracting structured databases from publications describing genome-wide association studies. Our results represent a step towards using machine reading algorithms to help human curators synthesize knowledge in the biomedical literature, helping make GWAS research faster and more accurate.

## Methods

### Detailed description of the GWASkb system

The system used to create GWASkb is implemented in Python on top of the Snorkel IE framework.^10^ Snorkel provides utilities for parsing XML documents and training machine learning classifiers. The GWASkb system extends the parsers/classifiers in Snorkel and applies them to the GWAS extraction task. Below, we provide additional details on the various components of the system.

To identify simple phenotypes, we start by parsing paper titles and abstracts and generate candidates from the EFO, Snomed, and Mesh ontologies. We use 11 labeling functions (LFs), which include the following: is the mention in the title; is the mention less than five characters; does the mention contain nouns; is the mention in the first half of the sentence, etc. We include the full list of LFs in Supplementary Note 6. The high-level phenotype is the set of three highest scoring mentions exceeding a user-specified score threshold or the single highest mention if none exceeds the threshold; this enables us to handle multiple valid phenotypes.

To identify precise phenotypes, we start by only parsing tables and generate candidates from cells whose header contains the words “phenotype”, “trait”, or “outcome”. Candidate *p* values are generated by matching a regular expression; candidate relations consist of horizontally aligned phenotype and *p* value candidates. We use three LFs (provided in Supplementary Note 6): is the candidate mostly a number; is the header of the cell (indicating it is in a phenotype column) very long; does the mention contain words referring to an rsid.

Next, we resolve acronyms by looking at the entire paper, including tables and the main natural language text in the body of the paper. We extract candidates from aligned pairs table cells, where one row is labeled “phenotype”, “trait”, or “description”, while the other is labeled “abbreviation”, “acronym”, or “phenotype”. We generate candidates from the main text using a regular expression. Our LFs, include the following: is the candidate all in caps; does the candidate match to the Snomed dictionary; does the acronym candidate consist of the letters of each word of the phenotype candidate; is one a prefix of the other; etc. The module for resolving abbreviations is linked in Supplementary Note 7.

Finally, we identify *p* values by again generating candidates from tables; SNP candidates are generated using a regular expression; *p* value candidates are ones that match one of three regular expressions (see Supplementary Note 8); candidate relations consist of horizontally aligned SNP and *p* value candidates (with at most one rsid per row). These candidates were accurate and we report them all.

### Mapping phenotypes across databases

In order to compare against GWAS Central and GWAS Catalog, we define mappings between GWASkb phenotypes and ones used in these two repositories. These mappings are tables with about 800 entries each that also indicate whether the mapping is fully or partially correct (e.g., “smoking behaviors” is less precise than “packs per day”). We define the latter as conceptually containing the precise label while also being not so broad as to be useless. See also our earlier discussion on high- and low-level phenotypes.

### Understanding the errors of GWASkb system components

Errors at the simple phenotype extraction stage mostly occur when the true phenotypes are not found in our candidate dictionaries (e.g., for the phenotype “genome-wide association study in bipolar patients,” we can only generate the candidate “bipolar disorder”). The second major source of error are phenotypes mentioned only in passing (e.g., the phenotype “high body fat is a risk for diabetes” when diabetes is not the phenotype whose association is being reported).

To estimate the precision of this module, we first restrict ourselves to (paper, rsid, and phenotype) relations produced by GWASkb that are also confirmed by an existing database, in the sense that the variant specified by the rsid occurs in some relation associated with the paper (but not necessarily one with the same phenotype). Then, we look at the fraction of these relations whose phenotype is also correct (at the approximate level). This gives precisions of 97% in the GWAS Catalog and 96% in GWAS Central.

Most errors at the precise phenotype extraction stage occur because we do not correctly resolve acronyms or because low-level phenotypes are not in tables (but rather only in text). Acronyms are not resolved most often because the shortened symbol is not clearly related to the full expression (e.g., CYS5 for Cysteine proteinase inhibitor 5 precursor), and they are presented in tables with confusing formatting. We estimate precision in the same way as for simple phenotypes, but this time, we require that phenotype agree fully. Precision was 73% in GWAS Central, the database with the most precise phenotypes. In GWAS Central, it was 82%.

To evaluate *p* value extraction accuracy, we labeled by hand 100 random relations and found that our rule-based extraction procedure had a precision of 98%. Errors occurred when *p* values referred to other entities in the row, such as haplotypes. Note also that oftentimes, variants and their *p* values are only provided in text but not in tables. This was the primary reason why we failed to report the rsid’s of 584 (15%) GWAS Catalog and 432 (14%) GWAS Central associations.

### Error analysis over 100 new relations

The 100 variants were not in the GWAS Catalog for one of the following reasons:

1. [44 variants]: Variants that are significant in one analysis cohort, but not in the combined meta-analysis. We believe such associations may still be useful in several applications, such as enrichment analysis. In order to make it easy to use these variants, we have extracted a set of meta-data for each variant (and described above); this meta-data can be used by researchers to determine the associations that are not significant in the meta-analysis.
2. [27 variants]: Variant is in the same locus as a more significant variant that is in also in the GWAS Catalog. However, the LD between these two variants is weak. Even though two variants are in same locus (i.e., within the same genomic region) they may not be in strong LD. We found this happened quite often; we validated our estimated LD numbers (these were derived from the 1000 Genomes dataset) with an online tool from the NIH. In our analysis we used *r*^2^ < 0.5 in the most precise population available for the study (e.g., CEU, EUR, and ALL) as a threshold for what constitutes weak LD. When the LD is weak according to both our estimates and the NIH tool, we believe that cataloguing our proposed association would be useful to researchers.
3. [9 variants]: Variant is in the same locus as a more significant variant that is in also in the GWAS Catalog. The LD between these two variants is strong. These variants may not be useful as the variants that are in weak LD. However, including them may be still useful in some uses cases, because the LD cutoff for what constitutes a strongly correlated variant may change in the future. Collecting these variants allows users to later select the subset of the data that is relevant to their needs.
4. [8 variants]: Variant appears in previous paper, but is also found to be significant in this paper. The variant was found to be significant in an earlier study, and in the discovery stage of the current study, but not in its meta-analysis stage. The GWAS Catalog guidelines indicate that such variants should be included, but we found cases when they were not.
5. [5 variants]: Variant appears in previous paper, but is not found to be significant in this paper. The variant was found to be significant in an earlier study, but not the discovery stage of the current study, hence it was correctly not included in the GWAS Catalog.
6. [7 variants]: GWASkb extraction error. We extracted an incorrect phenotype for these variants.

Most of the above variants have been excluded from the GWAS Catalog for scientific reasons. However, we recommend a large number of these variants for inclusion in a broader database, because they are still relevant to researchers. These include, 8 variants that have been replicated from a previous study, 27 variants that are in the same locus as a GWAS Catalog variant, but whose LD is weak (35 variants in total). In addition, 44 variants that have not been replicated in a meta-analysis and 9 variants that are in LD with GWAS Catalog variants at *r*^2^ ≥ 0.5 (50 variants in total) may also be useful in a limited number of applications, as described above. The remaining 12 variants are not worth curating, and represent a GWASkb error. See Supplementary Note 4 for further detail.

Overall, these are the key takeaways of the analysis:

- Our inclusion criterion is less stringent than that of the GWAS Catalog, but would be comparable to that of some other human-curated databases, such as GWAS Central.
- Providing an extended set of associations—a large part of which is valid and can be efficiently verified—has the potential to assist curators. The additional variants not in the GWAS Catalog can still be useful for certain analysis, but researchers need to use their judgment before using them.
- Our system also produces a small number of errors. For this reason, we recommend that all automated extractions be validated, though we expect the validation process to be much faster than discovery.

Estimating the precision of GWASkb

We estimate our overall precision at 94% relative to the output specifications of our system. Of the 6422, associations reported by our system, we consider 3463 to be correct because we could confirm them in an existing database (GWAS Catalog or GWAS Central). We estimate the error rate on the other 2959 relations to be between 12% (incorrect and repeat relations; this corresponds to GWASkb specifications) and 53% (when adding the 44 variants not confirmed in the meta-analysis; these are the set of associations in which we would have the least confidence), for an estimated total precision of 78–94% over the 6422 reported relations.

## Supplementary information

Supplementary Information

Peer Review File

Source Data

## Data Availability

The complete datasets and code used in the current study are available in the gwaskb repository, accessible at https://github.com/kuleshov/gwaskb. The resulting knowledge base, GwasKB, is also accessible via a web portal at http://gwaskb.stanford.edu/. All other data are contained within the article and its supplementary information (the source data folder contains source code, raw input data including papers and ontologies, extra figures, extra Jupyter analysis notebooks; see Supplementary Note 5).

## Code availability

The complete datasets and code used in the current study are available in the gwaskb repository, accessible at https://github.com/kuleshov/gwaskb, which includes full documentation for running the code and reproducing results.
