# Supplementary material for: A machine-compiled database of genome-wide association studies
Source: Nat Commun. 2019 Jul 26;10:3341. doi: 10.1038/s41467-019-11026-x (PMC6659642; doi:10.1038/s41467-019-11026-x)
Supplement: Supplementary file 1 — Supplementary Information [file 41467_2019_11026_MOESM1_ESM.pdf]

Supplementary Information Cover Page

Title: A Machine-Compiled Database of Genome-Wide Association Studies

Author: Kuleshov, et al.

A Machine-Compiled Database of Genome-Wide Association Studies

Supplementary Materials

Table of Contents

1. Supplementary Figures 3

2. Supplementary Tables 7

3. Supplementary Notes 8

3.1. Supplementary Note 1: Data Programming 8

3.2. Supplementary Note 2: Analysis of Novel Variants 10

3.2.(a) Linkage Disequilibrium 10

3.2.(b) Pathway Analysis 11

3.2.(c) Distribution of Effect Sizes 12

3.3 Supplementary Note 3: GWAS Study Format 13

3.4. Supplementary Note 4: Analysis of New Variants Found as Compared to GWAS Catalog 14

3.5. Supplementary Note 5: Data Download 16

3.6. Supplementary Note 6: Labeling Functions 16

3.7. Supplementary Note 7: Acronym Resolution 21

3.8. Supplementary Note 8: P-value Regular Expressions 22

3.9. Supplementary Note 9: Extracted Meta-Data 22

3.10. Supplementary Note 10: Website Interface 24

3.11. Supplementary Note 11: Github Table of Contents 24

1. Supplementary Figures

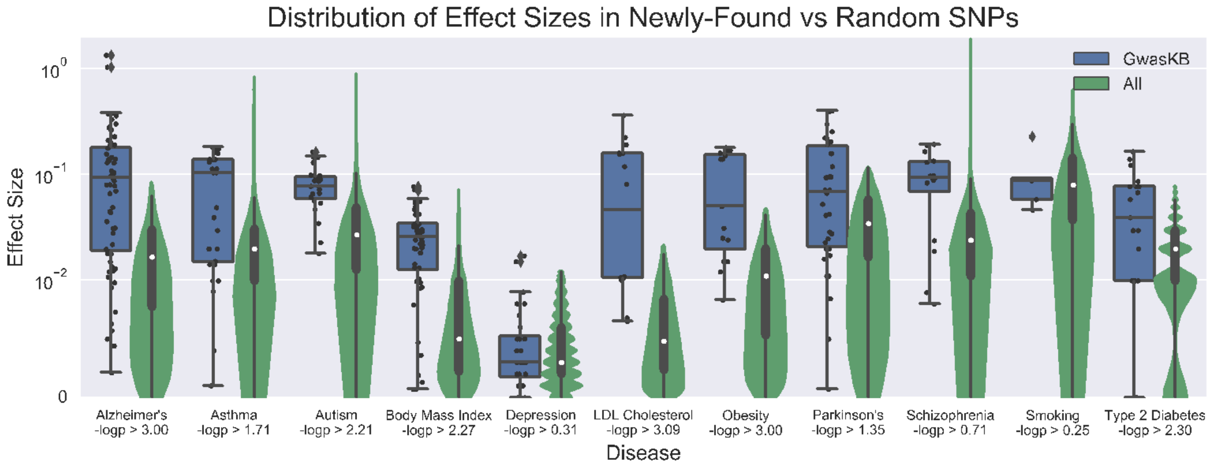

Supplementary Figure 1: Visualizing the effect sizes of variants identified by GwasKB.

We compare the distribution of effect sizes (absolute values of beta coefficients or log odds ratios; data from LD Hub) of variants identified by GwasKB (blue) to that of all variants (green) for multiple traits. We only look at novel GwasKB variants not present in existing human-curated repositories. In the boxplots, center lines represent medians, the box boundaries span the interquartile range, and the whiskers extend to the minimum and maximum observations excluding statistical outliers.

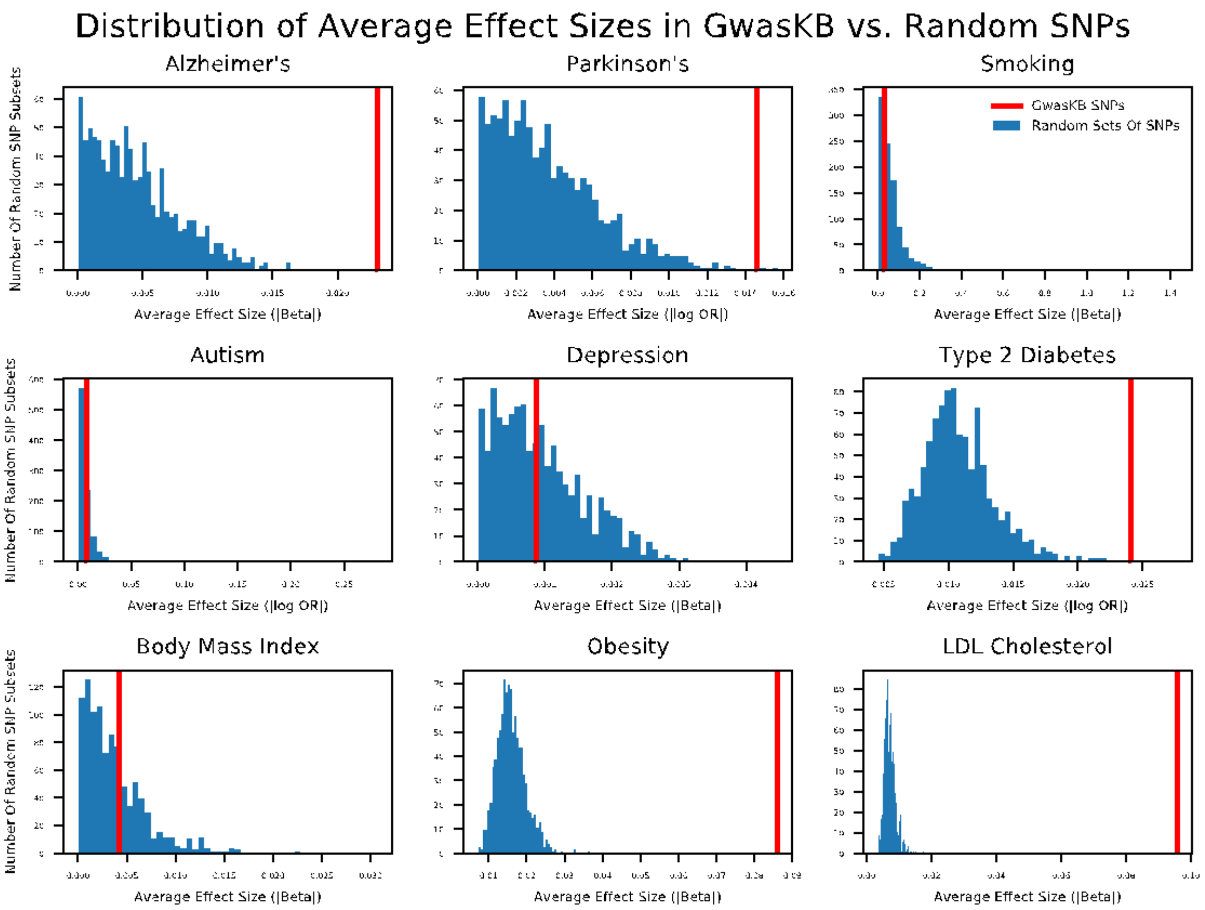

Supplementary Figure 2: Visualizing the effect sizes of variants identified by GwasKB.

We subsample 1000 random sets of variants with the same number of elements as the set of GwasKB SNPs for a given disease; the average effect size of GwasKB variants (red) is higher than that of the random subsets (blue). We only look at novel GwasKB variants not present in existing human-curated repositories.

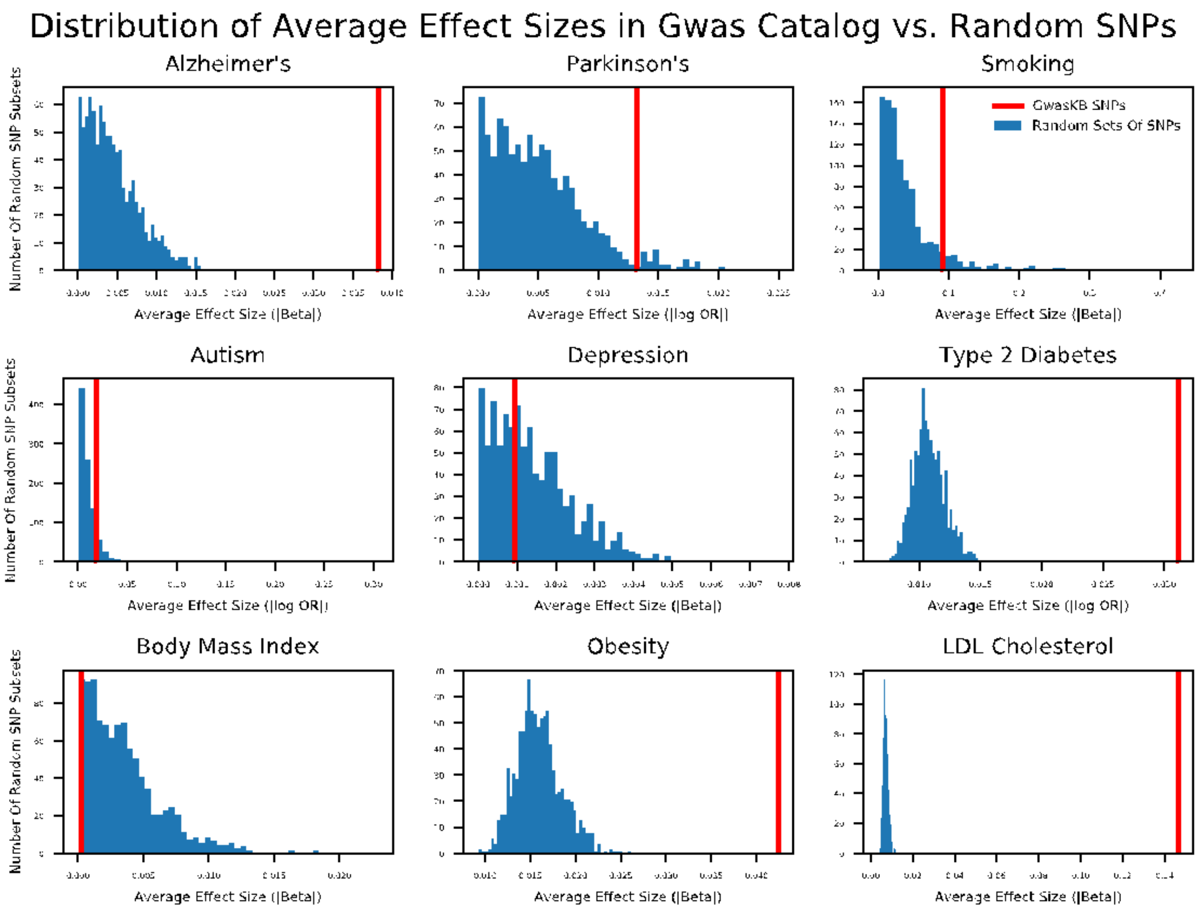

Supplementary Figure 3: Visualizing the effect sizes of variants GWAS hits reported in the GWAS Catalog.

We subsample 1000 random sets of variants with the same number of elements as the set of GWAS Catalog SNPs for a given disease; the average effect size of GWAS Catalog variants (red) is higher than that of the random subsets (blue).

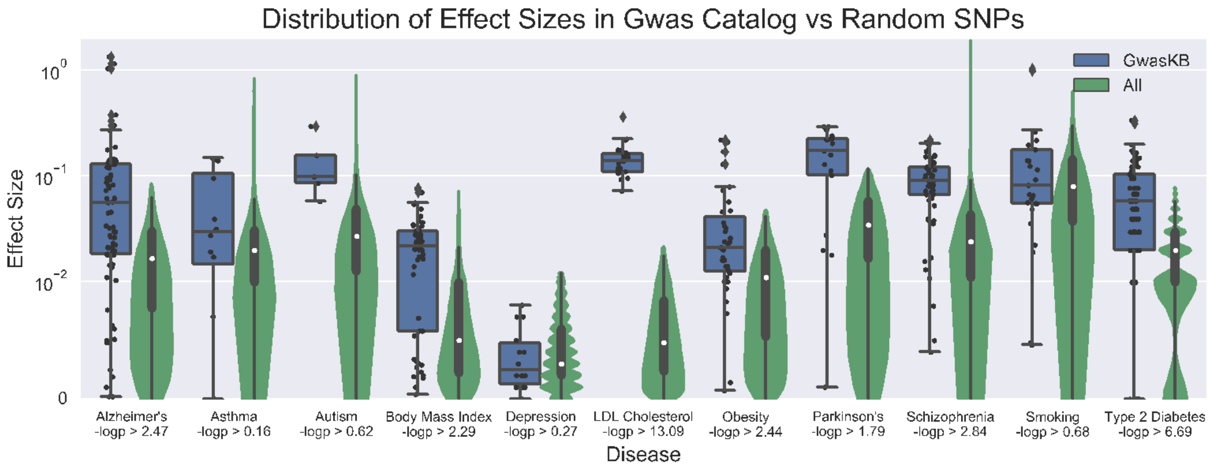

Supplementary Figure 4: Visualizing the effect sizes of variants in the GWAS Catalog.

We compare the distribution of effect sizes (absolute values of beta coefficients or log odds ratios; data from LD Hub) of variants present in the GWAS Catalog (blue) to that of all variants (green) for multiple traits. The differences between the distributions are comparable to ones observed for GWASkb variants.

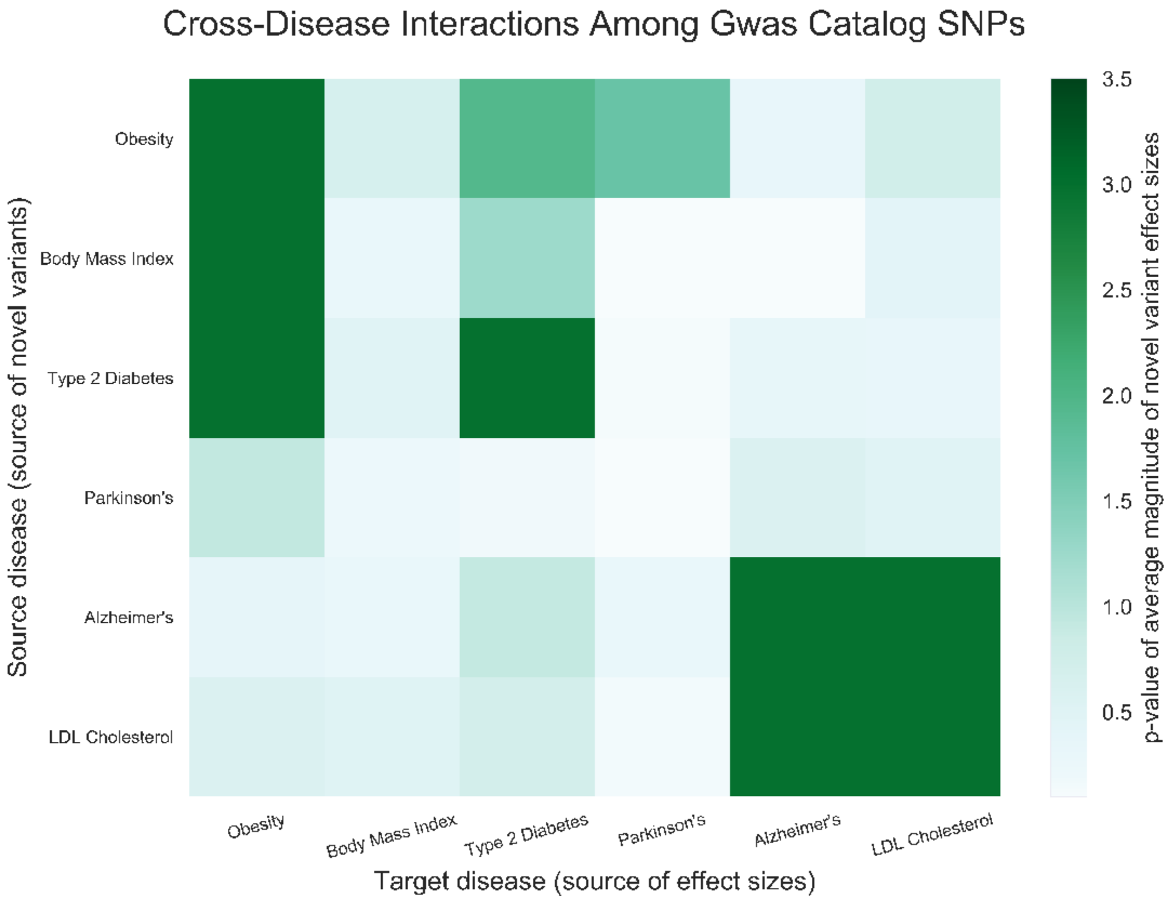

Supplementary Figure 5: Visualizing the effects of variants identified in the GWAS Catalog for pairs of related phenotypes.

For each pair of phenotypes, we compute the average absolute effect size of GWAS Catalog SNPs from the first phenotype (left) using summary statistics from the second phenotype (right; summary statistics were obtained from the LD hub). The heat map displays the log-probability of observing an equal or greater effect size by sampling random variants (we thus compute p-values using a one-sided permutation test). The heatmap displays the same blocks of related diseases as when we used GwasKB variants.

2. Supplementary Tables

cognitive disease	5.51E-37
dementia	9.29E-28
neurodegenerative disease	3.89E-27
Alzheimer's disease	3.48E-26
tauopathy	1.11E-25
schizophrenia	1.1E-19
psychotic disease	1.17E-19
peripheral nervous system disease	4.4E-18
cerebral arterial disease	4.79E-18
polyneuropathy	1E-17
vascular dementia	1.14E-17
neuropathy	5.84E-16
congenital anemia	1.49E-15
Lewy body disease	1.73E-15
amyloidosis	3.9E-15
motor neuron disease	4.93E-15
hereditary degenerative disease of central nervous system	1E-14
congenital hypoplastic anemia	2.38E-14
leukodystrophy	3.19E-14
neuromuscular disease	5.25E-14

Supplementary Table 1: Disease Ontology (DO) terms that have been found using the GREAT tool to be significantly enriched among variants determined by GwasKB to be associated with neurodegenerative diseases (27 traits, including Autism, Alzheimer's, Parkinson's).

The DO terms are also highly related to neurodegenerative diseases, which provides external validation for the relevance of the GwasKB variants.

disease by infectious agent	1.65E-31
viral infectious disease	2.58E-23
autoimmune disease	6.44E-21
demyelinating disease	1.36E-17
DNA virus infectious disease	1.52E-17
demyelinating disease of central nervous system	6.83E-17
RNA virus infectious disease	8.82E-17
multiple sclerosis	2.1E-16
bone marrow disease	2.3E-16
dsDNA virus infectious disease	4.26E-16

congenital anemia	2.03E-15
aplastic anemia	3.27E-15
Plasmodium falciparum malaria	4.5E-15
anemia	1.96E-13
thyroid gland disease	3.7E-13
Herpesviridae infectious disease	4.37E-13
parasitic infectious disease	6.93E-13
Hodgkin's lymphoma	8.31E-13
Diamond-Blackfan anemia	1.04E-12
bone inflammation disease	1.86E-12

Supplementary Table 2: Disease Ontology (DO) terms that have been found using the GREAT tool to be significantly enriched among variants determined by GwasKB to be associated with auto-immune diseases (23 traits, including Diabetes, Arthritis, Lupus, etc.).

The DO terms are also highly related to auto-immune diseases, which provides external validation for the relevance of the GwasKB variants.

3. Supplementary Notes

3.1. Supplementary Note 1: Data Programming

One of the most significant bottlenecks in developing machine learning-based applications today is the challenge of collecting large sets of hand-labeled training data. Especially with today's complex machine learning models—for example, deep neural networks, which sometimes have hundreds of millions of free parameters—increasingly massive labeled training sets are required. Moreover, such training sets are completely static and cannot be modified or upgraded to accommodate new or changed modeling objectives.

Data programming is a newly-proposed paradigm for training models using higher-level, weaker supervision to avoid this bottleneck. In data programming, users write a set of labeling functions, which are simply black-box functions that label data points, thus generating large, noisy sets of labels. These labeling functions can express and subsume a wide variety of heuristic approaches such as distant supervision—where an external knowledge base is used to label data points—regular expression patterns, heuristic rules, and more. These labeling functions are assumed to be

better than random, but otherwise may have arbitrary accuracies, may overlap, and may conflict. The core modeling task in data programming is to estimate the accuracies of the labeling functions so as to synthesize the noisy labels they generate in a statistically sound way, using only unlabeled data.

In the standard data programming setting, we model each labeling function as a noisy "voter" with some greater-than-random accuracy, and which makes errors that are uncorrelated with the other labeling functions. In other words, we assume that the labeling functions are all conditionally independent given the (unobserved) true label. Considering the binary classification setting for simplicity, let $\lambda_{i,j} \in \{-1,0,1\}$ be the label given by the jth labeling function to the ith data point. Our independence assumption is thus stated as $\lambda_{i,j} \perp \lambda_{i,k\neq j} | y_i$, and for each labeling function our goal is to learn a weight representing its probability of providing a label (versus abstaining), $\theta_j^{Lab} \propto P(\lambda_{i,j} \neq 0)$, and a weight representing its probability of labeling correctly, $\theta_j^{Acc} \propto P\left(\lambda_{i,j} = y_i | \lambda_{i,j} \neq 0\right)$. This defines a simple generative model of a noisy labeling process described by the provided labeling functions.

To learn the resulting *generative labeling model*, we first apply all the labeling functions to the unlabeled data points, resulting in the *label matrix* λ. We then encode the model $p_\theta(\lambda, y)$ as a factor graph. We start by defining two factor types representing the labeling propensity and accuracy of the labeling functions:

$$\phi_{i,j}^{Lab}(\lambda) = \mathbb{1}\{\lambda_{i,j} \neq 0\}$$

(1)

$$\phi_{i,j}^{Acc}(\lambda, y) = \mathbb{1}\{\lambda_{i,j} = y_i\} \quad (2)$$

For a given data point x_i, we define the concatenated vector of these factors for all the labeling functions j=1,..,M as $\phi_i(\lambda, y)$, and the corresponding vector of parameters θ. This then defines our model:

$$p_\theta(\lambda, y) = Z_\theta^{-1} \exp(\sum_{i=1}^{N} \theta^T \phi_i(\lambda, y)) \quad (3)$$

where Z_θ is a normalizing constant. To learn this model, *without* access to the true labels y, we minimize the negative log marginal likelihood given the observed label matrix λ:

$$\hat{\theta} = argmin_\theta - \log\left(\sum_{y'} p_\theta(\lambda, y')\right) \quad (4)$$

We can optimize this objective by interleaving stochastic gradient descent steps with sampling ones, similar to contrastive divergence. We perform this gradient descent and sampling procedure using the Numbskull library, a Python NUMBA-based Gibbs sampler, within the Snorkel framework for data programming.

The predictions of the trained generative labeling model, $\tilde{y} = p_{\hat{\theta}}(y|\lambda)$ can then either be used directly as predictions for the data points, or as probabilistic training labels to train a second discriminative model. In our setting, the coverage of the labeling functions is sufficient such that we directly apply these probabilistic labels as predictions. In either case, by adding more unlabeled data, we can increase the performance of both models with similar asymptotic scaling as if we were using labeled training data; for more specific results, see the work by Ratner et al.

3.2. Supplementary Note 2: Analysis of Novel Variants

In this section, we give more details on the procedure we used to analyze the biological function of the novel variants identified by GwasKB.

3.2.(a) Linkage Disequilibrium

We estimate linkage disequilibrium (LD) between variants using the PLINK software pacakge based on data from the Thousand Genomes (1000G) Project. The full set of scripts used to obtain the statistics are available in the GwasKB Github repository at the following link:

https://github.com/kuleshov/gwaskb/blob/338f4f6cfb3b6c79a312e0ab901abff038134a45/notebooks/bio-analysis/enrichment/enrichment.ipynb

We also include relevant code snippets below.

For each novel variant, we obtain a 1Mbp window of the 1000G data centered at the variant:

```
tabix -fh ftp://ftp.1000genomes.ebi.ac.uk/vol1/ftp/release/20100804/ALL.2of4intersection.20100804.genotypes.vcf.gz <region> > genotypes.vcf
```

Next, we use vcftools to output the variants in PLINK format:

```
./vcftools --vcf ~/genotypes.vcf --plink-tped --out plinkformat
```

Finally, we convert the output to

```
plink --bfile delete --r2 --ld-snp-list list.txt --ld-window-kb 10000 --ld-window 99999 --ld-window-r2 0 --out ld_results
```

where we compute the LD to known SNPs.

This procedure creates files with r2 distances to known variants surrounding every novel variant.

Next, we use custom scripts (see the aforementioned Github notebook) to compute the closest known variant to each new SNP. We then iterate through all of the new SNPs and keep ones that (1) are in the 1000G dataset, (2) their closest known SNP is in the 1000G dataset, (3) the max r^2 score between the new variant and known variants from the same paper is below a user-specified threshold (0.5) in our experiments.

3.2.(b) Pathway Analysis

Two major subsets of phenotypes were formed based on the predicted trait of each variant: neuro-degenerative diseases (ND) and autoimmune diseases (AU). We hand selected traits known to be linked to either class and filtered the variants based on these sets of traits, ultimately leading to 283 ND variants and 155 AU variants after further LD filtering. The list of phenotypes defined as neuro-degenerative diseases includes Alzheimer's, Parkinson's, schizophrenia, dementia, autism, stroke, epilepsy, neurodegeneration, neuroblastoma, amyotrophic lateral sclerosis, atherosclerosis, and multiple sclerosis. Similarly, the list of phenotypes defined as autoimmune diseases includes type 2 diabetes, obesity, lupus, celiac disease, Crohn's disease, immunoglobulin, rheumatoid arthritis, psoriatic arthritis, and osteoarthritis.

To obtain the two sets of genes with preferential brain expression and preferential blood expression, we used the dataset of genes available on GTEx Portal (https://gtexportal.org/home/datasets). The sets of genes were produced by selecting genes linked with brain or blood tissues using a mean RPKM threshold of 150.

To determine that variants associated with ND diseases occur significantly more within 200Kbp of genes with preferential brain expression and that AU disease linked variants are found more frequently near blood genes, we performed a chi-squared test for independence. The corresponding contingency table consisted of two rows: one each for ND variants and for AU variants, and three columns: found near a gene with preferential brain expression, found near a gene with preferential blood expression, and other which includes being found near genes expressed in both brain and blood tissues or near neither. The contingency table is found below.

Variant/Gene Type	Found near Brain Genes	Found near Blood Genes	Other	Total
ND Variants	25	7	251	283
AU Variants	5	10	140	155
Total	30	17	391	438

3.2.(c) Distribution of Effect Sizes

We analyzed the degree to which novel variants impacted their predicted phenotypes on the 11 most frequent traits from our dataset linked to either ND or AU diseases. The summary statistics for each disease, which includes various SNPs and known effect sizes, were obtained through relevant studies from the LD Hub project, and a specific list of the 11 traits and their corresponding summary statistics links can be found below.

Disease	Summary Statistics Link
Alzheimer's	http://web.pasteur-lille.fr/en/recherche/u744/igap/igap_download.php
Parkinson's	https://www.ncbi.nlm.nih.gov/projects/SNP/gViewer/gView.cgi?aid=2868
Schizophrenia	https://www.med.unc.edu/pgc/results-and-downloads
Autism	https://www.med.unc.edu/pgc/results-and-downloads
Smoking	https://www.med.unc.edu/pgc/results-and-downloads
Depression	https://www.thessgac.org/data
Type 2 Diabetes	http://diagram-consortium.org/downloads.html

Body Mass Index	http://portals.broadinstitute.org/collaboration/giant/index.php/GIANT_consortium_data_files
Obesity	http://portals.broadinstitute.org/collaboration/giant/index.php/GIANT_consortium_data_files
Asthma	http://www.cng.fr/gabriel/results.html
LDL Cholesterol	http://csg.sph.umich.edu//abecasis/public/lipids2013/

An effect size is a quantitative measure of a single variant's impact on a particular phenotype. For the LD Hub studies, effect sizes are measured using either a beta statistic or an odds ratio. The beta statistic is centered around zero which indicates the variant has no effect on the phenotype. Similarly, the odds ratio indicates variant correlations with phenotype expression; we use the log odds ratio in our experiments, which is also centered around zero. We look the absolute values of both measures in most experiments.

To examine cross-disease correlations of effect sizes for each pair of diseases (including the same diseases), we performed a permutation test to compute the probability of observing the absolute average effect size of the novel variants within a random set of SNPs. Essentially, we compared the average effect size of the set of variants linked with the first disease using the summary statistics for the second disease with the average effect sizes of multiple random samples from the distribution of all variants with a known effect size for the second disease.

For each disease, we also compared the distribution of the novel variants to the distribution of all SNPs with known effect sizes for that disease from the summary statistics using the nonparametric Kolomogorov-Smirnov (KS) statistical test. The KS test provides a useful method for comparing whether two distributions differ. Results from performing the test indicated that for many of the diseases analyzed, the novel SNPs do follow a different distribution from the general distribution of all SNPs.

Finally, we combined both tests to obtain a p-value on the null hypothesis that novel and known variants originate from the same distribution. We used the formula $\log p_{joint} < \min(0.5*\min(\log p_1, \log p_2), \max(\log p_1, \log p_2))$, where p_1, p_2 are the KS and permutation test p-values, respectively.

3.3 Supplementary Note 3: GWAS Study Format

Genome-wide association studies (GWAS) are widely used for measuring the effects of genetic mutations on human traits. These are typically large case-control studies in which hundreds of thousands or more variants are measured in each study participant. The variants that are significantly enriched in one cohort versus the other are reported.

In order to reduce the frequency of false positives, GWAS often consist of a discovery stage that is then followed by one or more replication stages. The final results are obtained from a meta-analysis of all the cohorts. The power of a GWAS is a function of both the number of variants and the number of participants in the study. In addition, other information, such as the ethnicity of the study or the statistical methodology used are important for understanding the results of a GWAS.

Our work focuses on extracting triples of (variant, phenotype, p-value). A notable limitation of our system is that it does not output study sizes or populations. In the current version of GwasKB, this information needs to be extracted manually; however, this only needs to be done once per paper (rather than for every association).

3.4. Supplementary Note 4: Analysis of New Variants Found as Compared to GWAS Catalog

We collected and annotated a randomly-sampled set of 100 associations not present in the GWAS Catalog, that have been pre-filtered based on LD. We annotated each association with the following information:

- The reason for which the association is not in the GWAS Catalog.
- Whether the association was excluded due to curator error.
- Whether we recommend it for inclusion in a curated database. We base this recommendation on whether we think there are situations in which the association will be useful to researchers.

The 100 variants were not in the GWAS Catalog for one of the following reasons:

1. **[44 variants]** Variants that are significant in one analysis cohort, but not in the combined meta-analysis. We believe such associations may still be useful in several applications, such as enrichment analysis. In order to make it easy to use these variants, we have extracted a set of meta-data for each variant (and described above); this meta-data can be used by researchers to determine the associations that are not significant in the meta-analysis.
2. **[27 variants]** Variant is in the same locus as a more significant variant that is in also in the GWAS Catalog. However, the LD between these two variants is weak. Even though two variants are in same locus (i.e. within the same genomic region) they may not be in strong LD. We found this happened quite often; we validated our estimated LD numbers (these were derived from the 1000 Genomes dataset) with an online tool from the NIH. In our analysis we used $r^2 < 0.5$ in the most precise population available for the study (e.g. CEU, EUR, ALL) as a threshold for what constitutes weak LD. When the LD is weak according to both our estimates and the NIH tool, we believe that cataloguing our proposed association would be useful to researchers.
3. **[9 variants]** Variant is in the same locus as a more significant variant that is in also in the GWAS Catalog. The LD between these two variants is strong. These variants may not be useful as the variants that are in weak LD. However, including them may be still useful in some uses cases, because the LD cutoff for what constitutes a strongly correlated variant may change in the future. Collecting these variants allows users to later select the subset of the data that is relevant to their needs.
4. **[8 variants]** Variant appears in previous paper, but is also found to be significant in this paper.The variant was found to be significant in an earlier study, and in the discovery stage of the current study, but not in its meta-analysis stage. The GWAS Catalog guidelines indicate that such variants should be included, but we found cases when they were not.
5. **[5 variants]** Variant appears in previous paper, but is not found to be significant in this paper. The variant was found to be significant in an earlier study, but not the discovery stage of the current study, hence it was correctly not included in the GWAS Catalog.
6. **[7 variants]** GWASkb extraction error. Our system extracted an incorrect phenotype for these variants.

Most of the above variants have been excluded from the GWAS Catalog for scientific reasons. However, we recommend a large number of these variants for inclusion in a broader database, because they are still relevant to researchers. These include 8 variants that have been replicated from a previous study, 27 variants that are in the same locus as a GWAS Catalog variant, but whose LD is weak (35 variants in total). In addition, 44 variants that have not been replicated in a meta-analysis and 9 variants that are in LD with GWAS Catalog variants at $r2 \geq 0.5$ (50 variants in total) may also be useful in a limited number of applications, as described above. The remaining 12 variants are not worth curating, and represent a GWASkb error.

More broadly, we emphasize that our goal in the proposed approach was to give researchers increased flexibility with respect to the particular annotation guidelines used. In our automated approach, if researchers wish to populate the database according to different annotation guidelines, this can be accomplished simply by changing a minimal amount of code and re-running our pipeline- rather than needing to manually re-annotate an entire corpus.

The full set of annotations of this random sample is included in the Supplementary Files (https://github.com/kuleshov/gwaskb/blob/master/annotations/not_in_gwasc.xlsx).

3.5. Supplementary Note 5: Data Download

The raw data for GWASkb can be downloaded as a zipfile from the following Google Drive link:

https://drive.google.com/file/d/1DX17UCztwXtB3PxKLQd2waUBJdSdNJDc/view?usp=sharing

See the Github repository README at for more information:
https://github.com/kuleshov/gwaskb/blob/master/README.md

3.6. Supplementary Note 6: Labeling Functions

The following labeling functions can also be found and viewed in context in the Github repository:

https://github.com/kuleshov/gwaskb/blob/master/notebooks/lfs.py

```
import re
from bs4 import BeautifulSoup as soup
from snorkel.lf_helpers import *
import string
from nltk.stem import PorterStemmer
from nltk.corpus import stopwords as nltk_stopwords
from db.kb import KnowledgeBase


##### ACRONYM EXTRACTION
# AcroPhenRel = candidate_subclass('AcroPhenRel', ['acro','phen'])

### LFs for extraction from tables
def LF1_digits(m):
    txt = m[1].get_span()
    frac_num = len([ch for ch in txt if ch.isdigit()]) / float(len(txt))
    return -1 if frac_num > 0.5 else +1

def LF1_short(m):
    txt = m[1].get_span()
    return -1 if len(txt) < 5 else 0


### LFs for extraction from text
# helper fns
def r2id(r):
    doc_id = r[0].parent.document.name
    str1, str2 = r[0].get_span(), r[1].get_span()
    acro = str1[1:-1]
    phen = str2.split(' (')[0]
    return (doc_id, acro, phen)

# positive LFs
def LF_acro_matches(m):
    _, acro, phen = r2id(m)
    words = phen.strip().split()
    if len(acro) == len(words):
        w_acro = ''.join([w[0] for w in words])
        if w_acro.lower() == acro.lower():
            return +1
    return 0

def LF_acro_matches_with_dashes(m):
    _, acro, phen = r2id(m)
    words = re.split(' |-', phen)
    if len(acro) == len(words) and len(words) > 0:
        w_acro = ''.join([w[0] for w in words if w])
        if w_acro.lower() == acro.lower():
            return +1
    return 0
```

```
def LF_acro_first_letter(m):
    _, acro, phen = r2id(m)
    if not any(l.islower() for l in phen): return 0
    words = phen.strip().split()
    if len(acro) <= len(words):
        if words[0].lower() == acro[0].lower():
            return +1
    return 0

def LF_acro_prefix(m):
    _, acro, phen = r2id(m)
    phen = phen.replace('-', '')
    if phen[:2].lower() == acro[:2].lower():
        return +5
    return 0

def LF_acro_matches_last_letters(m):
    _, acro, phen = r2id(m)
    words = phen.strip().split()
    prev_words = left_text(m[1], window=1) + words
    w_prev_acro = ''.join([w[0] for w in prev_words])
    if w_prev_acro.lower() == acro.lower(): return 0
    for r in (1,2):
        new_acro = acro[r:]
        if len(new_acro) < 3: continue
        if len(new_acro) == len(words):
            w_acro = ''.join([w[0] for w in words])
            if w_acro.lower() == new_acro.lower():
                return +1
    return 0

def LF_full_cell(m):
    """If only phrase in cell is A B C (XYZ), then it's correct"""
    if not hasattr(m[1].parent, 'cell'): return 0
    _, acro, phen = r2id(m)
    cell = m[1].parent.cell
    txt_cell = soup(cell.text).text if cell.text is not None else ''
    txt_span = m[1].get_span()
    return 1 if cell.text == txt_span or txt_cell == txt_span else 0

def LF_start(m):
    punc = ',.;!?()\'"'
    if hasattr(m[1].parent, 'cell'): return 0 # this is only for when we're
within a sentence
    if m[1].get_word_start() == 0 or any(c in punc for c in left_text(m[1],
window=1)):
        _, acro, phen = r2id(m)
        if phen[0].lower() == acro[0].lower():
            return +1
    return 0

# negative LFs
def LF_digits(m):
    txt = m[1].get_span()
    frac_num = len([ch for ch in txt if ch.isdigit()]) / float(len(txt))
```

```
    return -1 if frac_num > 0.5 else +1

def LF_short(m):
    _, acro, phen = r2id(m)
    return -1 if len(acro) == 1 else 0

def LF_lc(m):
    _, acro, phen = r2id(m)
    return -1 if all(l.islower() for l in acro) else 0

def LF_uc(m):
    _, acro, phen = r2id(m)
    return -2 if not any(l.islower() for l in phen) else 0

def LF_punc(m):
    _, acro, phen = r2id(m)
    punc = ',.;!?()'
    return -1 if any(c in punc for c in phen) else 0


### PHENOTYPE EXTRACTION (TEXT)
# Phenotype = candidate_subclass('SnorkelPhenotype', ['phenotype'])

punctuation = set(string.punctuation)
stemmer = PorterStemmer()

# load set of dictionary phenotypes
kb = KnowledgeBase()
phenotype_list = kb.get_phenotype_candidates()
phenotype_list = [phenotype for phenotype in phenotype_list]
phenotype_set = set(phenotype_list)

# load stopwords
with open('../data/phenotypes/snorkel/dicts/manual_stopwords.txt') as f:
    stopwords = {line.strip() for line in f}
stopwords.update(['analysis', 'age', 'drug', 'community', 'detect',
'activity', 'genome',
                  'genetic', 'phenotype', 'response', 'population',
'parameter', 'diagnosis',
                  'level', 'survival', 'maternal', 'paternal', 'clinical',
'joint', 'related',
                  'status', 'risk', 'protein', 'association', 'signal',
'pathway', 'genotype', 'scale',
                  'human', 'family', 'heart', 'general', 'chromosome',
'susceptibility', 'select',
                  'medical', 'system', 'trait', 'suggest', 'confirm',
'subclinical', 'receptor',
                  'class', 'adult', 'affecting', 'increase'])
stopwords.update(nltk_stopwords.words('english'))
stopwords = {stemmer.stem(word) for word in stopwords}

def get_phenotype(entity, stem=False):
    phenotype = entity.get_span()
    if stem: phenotype = stemmer.stem(phenotype)
    return phenotype.lower()
```

```
def stem_list(L):
    return [stemmer.stem(l.lower()) for l in L]

def span(c):
    return c if isinstance(c, TemporarySpan) else c[-1]

def has_stopwords(m):
    txt = span(m).get_span()
    txt = ''.join(ch for ch in txt if ch not in punctuation)
    words = txt.lower().split()
    return True if all(word in stopwords for word in words) or \
                  all(stemmer.stem(word) in stopwords for word in words) or \
                  all(change_name(word) in stopwords for word in words) else
False

# positive LFs
def LF_first_sentence(m):
    return +15 if span(m).parent.position == 0 and not has_stopwords(m) else
0

def LF_from_regex(m):
    if span(m).parent.position == 0 and not regex_phen_matcher._f(span(m))
and not LF_bad_words(m): return +5
    else: return 0

def LF_with_acronym(m):
    post_txt = ''.join(right_text(m, attr='words', window=5))
    return +1 if re.search(r'\([A-Z]{2,4}\)', post_txt) else 0

def LF_many_words(m):
    return +1 if len(span(m).get_span().split()) >= 3 else 0

def LF_start_of_sentence(m):
    return +1 if m[0].get_word_start() <= 5 and not has_stopwords(m) and not
LF_no_nouns(m) else 0

def LF_first_mention_in_sentence(m):
    context_id = m[0].parent.document.name, m[0].parent.sentence.position
    other_pos = [c.get_word_start() for c in candidate_by_sent[context_id]]
    return +1 if m.get_word_start() == min(other_pos) else 0

# negative LFs
def LF_bad_words(m):
    bad_words = ['disease', 'single', 'map', 'genetic variation', '( p <']
    return -100 if any(span(m).get_span().lower().startswith(b) for b in
bad_words) else 0

def LF_short(m):
    txt = span(m).get_attrib_span('words', 3)
    return -50 if len(txt) < 5 else 0

def LF_no_nouns(m):
    return -10 if not any(t.startswith('NN') for t in
span(m).get_attrib_tokens('pos_tags')) else 0
```

```
def LF_not_first_sentences(m):
    return -1 if span(m).parent.position > 1 else 0

def LF_stopwords(m):
    return -50 if has_stopwords(m) else 0


### PHENOTYPE EXTRACTION (TABLES)
# RsidPhenRel = candidate_subclass('RsidPhenRel', ['rsid','phen'])

bad_words = ['rs number', 'rs id', 'rsid']

# negative LFs
def LF_number(m):
    txt = m[1].get_span()
    frac_num = len([ch for ch in txt if ch.isdigit()]) / float(len(txt))
    return -1 if len(txt) > 5 and frac_num > 0.4 or frac_num > 0.6 else 0

def LF_bad_phen_mentions(m):
    if cell_spans(m[1].parent.cell, m[1].parent.table, 'row'): return 0
    top_cells = get_aligned_cells(m[1].parent.cell, 'col', infer=True)
    top_cells = [cell for cell in top_cells]
    try:
        top_phrases = [phrase for cell in top_cells for phrase in
cell.phrases]
    except:
        for cell in top_cells:
            print cell, cell.phrases
    if not top_phrases: return 0
    matching_phrases = []
    for phrase in top_phrases:
        if any (phen_matcher._f_ngram(word) for word in phrase.text.split('
')):
            matching_phrases.append(phrase)
    small_matching_phrases = [phrase for phrase in matching_phrases if
len(phrase.text) <= 25]
    return -1 if not small_matching_phrases else 0

def LF_bad_word(m):
    txt = m[1].get_span()
    return -1 if any(word in txt for word in bad_words) else 0

# positive LFs
def LF_no_neg(m):
    return +1 if not any(LF(m) for LF in LF_tables_neg) else 0
```

3.7. Supplementary Note 7: Acronym Resolution

The code for resolving acronyms is included in a Jupyter notebook at the following link:

https://github.com/kuleshov/gwaskb/blob/master/notebooks/acronym-extraction.ipynb

3.8. Supplementary Note 8: P-value Regular Expressions

The code for identifying p-values is best viewed in a Jupyter notebook at the following link:

https://github.com/kuleshov/gwaskb/blob/master/notebooks/table-extraction.ipynb

They are also included here:

```
rgx1 = u'[1-
9]\d?[\xb7\.]?\d*[\s\u2009]*[\xd7\xb7\*][\s\u2009]*10[\s\u2009]*[-
\u2212\u2013\u2012][\s\u2009]*\d+'pval_rgx_matcher1 =
RegexMatchSpan(rgx=rgx1)rgx2 = u'[1-
9]\d?[\xb7\.]?\d*[\s\u2009]*[eE][\s\u2009]*[-
\u2212\u2013\u2012][\s\u2009]*\d+'pval_rgx_matcher2 =
RegexMatchSpan(rgx=rgx2)rgx3 = u'0\.0000+\d+'pval_rgx_matcher3 =
RegexMatchSpan(rgx=rgx3)pval_rgx_matcher = Union(pval_rgx_matcher1,
pval_rgx_matcher2, pval_rgx_matcher3)
```

3.9. Supplementary Note 9: Extracted Meta-Data

One of the main limitations of the current version of GWASkb is that it cannot determine in a fully automated way the cohort or the methodology used to identify an association. For example, it does not automatically report whether a particular p-value is from a discovery cohort, or from a meta-analysis, or from one of three ethnicities studied in the paper. In order to make it easier for users to obtain this information, we are extracting additional meta-data for each GWASkb p-value and providing it together with our set of associations. Specifically, we are extracting and providing in a separate file the contents of the first three cells in rows hierarchically above each p-value.

The following table illustrates the meta-data that we output. In this example, we report for each p-value (red) a string that describes its cohort (green).

Table 2. Discovery and follow-up genotyping results.

				Discovery		Follow-up		Combined			Annotation	
Chr	SNP	A1/A2	AF	Effect (se)	P-value	Effect (se)	P-value	Effect (se)	P-value	N	Location	Nearest Gene
2	rs17775170	A/G	0.27	−4.79E-02 (0.011)	5.36E-06	3.70E-03 (0.008)	6.218E-01	−1.38E-02 (0.006)	2.43E-02	7284	intronic	SLC9A2
2	rs2165179	A/G	0.33	−4.85E-02 (0.010)	1.47E-06	7.46E-03 (0.015)	6.280E-01	−3.17E-02 (0.008)	1.77E-04	7264	intronic	SCN3A

For example, the three p-values circled in red above would be associated with the following metadata (stored in a csv file):

doc_id	table_index	rows	cols	p-value	metadata
23056639	2	2	5	5.36e-06	[‘Discovery’, ‘P-value’]
23056639	2	2	7	6.218e-01	[‘Follow-up’, ‘P-value’]
23056639	2	2	9	2.43e-02	[‘Combined’, ‘P-value’]

More formally, this auxiliary meta-data is generated as follows. When a document is parsed, a Table object is created for each element in the document marked with <table> tags. A Table is composed of Cell objects that have a row start index, row end index, column start index, and column end index. Most Cells span only one column and run, but headers, for example, frequently span multiple rows, so we store row and column information in the more general format.

To find p-values in the tables, we use a regular expression that searches over the text in each cell. Where a p-value is found, we then iterate over the other cells in the table and save the text from any cell that overlaps with the column of the p-value, is in the top three rows of the table, and does not appear to be a p-value itself.

Ultimately, we would like to add a component to GWASkb that will predict the cohort associated with the p-value. In the meantime, this auxiliary dataset will be helpful to users that want to manually look up in which study stages a given association was significant. Combined with gold standard GWAS Catalog data, this dataset can also be used in follow-up work to train machine learning classifiers for directly predicting the stage of a specific study.

The file described here is included in the Github repository at the following link:

https://github.com/kuleshov/gwaskb/blob/master/notebooks/results/metadata/pval-rsid.metadata.tsv

3.10. Supplementary Note 10: Website Interface

Pasted below is a screenshot of the web interface we provide for searching GWASkb. This can be found at http://gwaskb.stanford.edu/

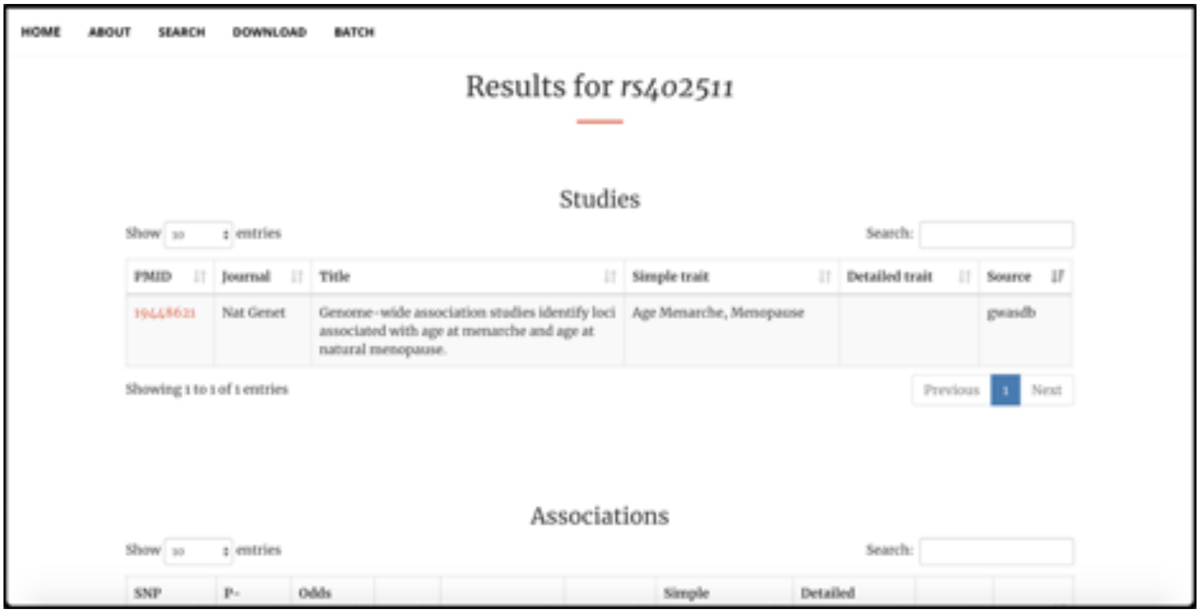

3.11. Supplementary Note 11: Github Table of Contents

Data and code referenced explicitly in the paper have direct links in the above sections. We include additional notebooks that walk through the step-by-step execution as well as intermediate calculations in the Github repository:

`https://github.com/kuleshov/gwaskb`

Copied below is a table of contents of that repository (shown with appropriate levels of nesting for ease of navigation) with accompanying brief descriptions:

Github Table of Contents (Directory Structure):

- **README.md**
- **annotations**: Manually annotated data
    - **not_in_gwasc.xlsx**: Manually annotated set of 100 relations extracted by GwasKB that were not in GWAS Catalog
- **data**: Datasets from which the knowledge base was compiled
    - **associations**: Human-curated associations against which we compare
    - **db**: Scripts to download and create the input database of publications
    - **phenotypes**: Scripts to generate phenotype ontology used by the system
- **notebooks**: Jupyter notebooks that walk us through how the system was used to generate the results
    - **bio-analysis**: Notebooks that reproduce the biological analysis performed in the paper
    - **lfs.py**: A Python file containing all labeling functions used
    - **results**: The main set of results produced by the machine curation system
        - **nb-output**: Intermediary output generated by each module (i.e., each notebook)
    - **metadata**: Metadata associated with extracted p-values
- **snorkel-tables**: The code for the version of Snorkel used in the project
- **src**: Source code of the components used on top of Snorkel
    - **crawler**: Scripts used to generate a database of papers as well as to crawl human-curated DBs
    - **extractor**: Modules that extend Snorkel to extracting GWAS-specific from the publications
- **results.md**: File documenting the output of the system
