# Supplementary material for: A machine-compiled database of genome-wide association studies
Source: Nat Commun. 2019 Jul 26;10:3341. doi: 10.1038/s41467-019-11026-x (PMC6659642; doi:10.1038/s41467-019-11026-x)
Supplement: Supplementary file 2 — Peer Review File [file 41467_2019_11026_MOESM2_ESM.pdf]

Reviewers' comments:

Reviewer #1 (Remarks to the Author):

The authors present a novel, data-programming approach, to machine-curation of GWAS associations. The results presented highlight that while such an approach should not totally replace the manual curation carried out by other resources, it has huge potential to lighten their workload and increase coverage. The authors themselves indeed suggest this joint approach in their well-balanced discussion.

The first assessment of their approach is the ability to recall previous, manually curated, associations from a set of 589 papers. Impressive performance is reported, with 69% of the associations from the GWAS catalog recalled with full accuracy in terms of the variant and phenotype, and 81% if allowing for approximately correct phenotypes. However, much of the workload of the manual curators in the GWAS catalog revolves around consistently and precisely defining the phenotypes using ontologies so that identical and/or related studies can easily be identified. The example in Table 2 of a fully accurate match with the GWAS catalog obviously describes the same phenotype but with a different text string: “Clozapine – Triglycerides”. Would other association describing this same phenotype be labelled with different text strings using their approach, making it harder to identify identical studies for meta-analysis?

Given the missed 19% manually curated associations are likely to be high quality and of interest to researchers, it would seem manual curation is still beneficial. It will be interesting to discuss how much time having the 81% already identified will save?

The more impressive result is the additional 2959 relations discovered in the set of papers that had already been curated for the GWAS catalog. Strong functional evidence is presented for many being accurate and worthy of inclusion in a GWAS database and having the results of GwasKB available for manual curators would clearly be beneficial. The authors have made great efforts to make the code and data openly accessible for such a purpose. The manual inspection of 100 randomly chosen examples suggested 60 should pass the criteria of the manual curators of resources such as GWAS catalog. Assuming this is the case, and extends more widely, it would seem an extraordinary miss-rate by the manual curation approach. Can the authors comment on why they think so many were missed by manual curation? Are the majority in LD with one of the manually curated variants from the same study and rejected by the manual curators as suggested in the subsequent section? Or is it simple, human error as suggested in the discussion. This is key to others assessing whether to incorporate GwasKB as part of their curation pipeline.

Reviewer #2 (Remarks to the Author):

This is a useful project and well written manuscript. The authors address an important issue, viz, the fact that not all GWAS hits reported in manuscripts make it into databases such as GWAS Catalog, which are widely used for many downstream analyses. Thus, the community is not taking full advantage of published data because of the difficulties in curating and integrating this data.

They propose a machine learning system based on the novel data-programming paradigm. The results are available on a website. For me, the most interesting part of the manuscript was the demonstration of the utility of the data programming paradigm for a medical data mining challenge. My main suggestion for the authors is that they should strive to provide a more detailed description and motivation of this methodology. They also do not really compare their work against previous work, and they should provide more of a discussion/comparison.

Minor comments.

1) The number of these new variants corresponds to about 20% of all open access associations recorded in the most up-to-date human-curated database, GWAS Catalog.

=> Is it true that GWAS catalog is the most up to date? What about GWASdb anbd GWAS Central etc.? Can the authors substantiate this claim? Or is the claim just based on the open access papers they examine?

2) With GwasKB, we restricted ourselves to open-access papers,

=> Have the authors tried to text-mine non-open access papers? Surely they have access to many in their university.

3) Finally, in the classification stage, we determine which of these candidates are actually correct relation mentions using a

135 machine learning classifier.

=> It would be better to say "...we predict..." rather than " we determine"

4) Finally, in the classification stage,we determine which of these candidates are actually correct relation mentions using a

machine learning classifier. We use a Naive Bayes classifier with a small number of hand-crafted features (between 4 and 12)

=> Did the authors tune the classifier for each type of phenotype? How did they do so and what were the criteria? They should provide more detail here. Also, they should

provide more detail as to how the best candidate features were chosen. I think that an example would be helpful, and the methodology should be described so that

in principle it could be reproduced based on the description in the methods section (I realize that much code is available as Python notebooks, which is excellent, but I still think

that the methods section needs to have a more precise overview of the methods).

5) line 260 We should note however that the vast majority of ND and AU variants were found far from coding regions.

=> I think you mean AI variants (not AU)

6) Examining the Effect Sizes of Novel GwasKB Variants

=> It is unclear to me why the authors perform this comparison since the estimated effect size is related to the p value cutoff they use and so their finding is an expected one.

7) In addition, past studies still need to be curated. An ideal solution appears to involve a combination of authors, machines, and curators.

=> It would be great if the authors would contact the maintainers of one of the current GWAS databases and agree to collaborate (Note: this reviewer is not connected with any such database).

Presumably the results of the authors' method could be used to improve the quality and scope of manual curation, thus providing the greatest utility to the community.

Reviewer #3 (Remarks to the Author):

Kuleshov et.al. have created a machine learning algorithm that extracts genetic associations from the literature. The associations are in the form of variant identifier (rsID), statistic of association (p-value) and the phenotype that the variant is associated (publication wide, high-level; and detailed). The authors test this extraction system on a set of open-access publications that have been included in the GWAS Catalog, a manually curated database of associations. They also do some validation using data from GWAS Central. The authors claim to extract 80% of known associations (those already in GWAS Cat/Central) and an additional approx three to four thousand associations not present in GWAS Cat/Central. The authors perform some analysis (pathway analysis, effect size analysis) on the 'new' associations to assert their functional relevance.

I should state at the outse that this reviewer is associated with the GWAS Catalog. Although this review will sound largely negative I want to emphasise that I am broadly positive about this work and view it is incredibly important and potentially very useful. I see the future of curation of this type of data as a collaboration between man and machine (which the authors also seem to). GWAS is particularly suited to machine extraction as the basic unit of the data is fairly stereotyped. Therefore this effort should be encouraged. I am very keen on seeing machine learning used to advance the public availability of data.

However, the paper broadly reads like it is written by machine learning computationalists who have not fully considered the underlying biology or the requirements of the downstream data consumer, who have not sufficiently investigated their reference data/truth set and who are overstating the utility of their product. If they re-write the paper, bearing in mind the points I outline below, GwasKB could constitute a step towards a very useful resource.

- Major point 1

Extraction of three pieces of information (rsID, p-value, phenotype) is minimal. There are many other data types the authors could extract, which they do mention they could expand to (e.g. effect size-beta/OR, SE, allele). Extraction of these would greatly enhance the ultility of the data, but I see this as a natural extension of the work and I'm not concerned at present that they are not extracted yet.

What I am concerned with, given how the authors present their data, is the lack meta-data provided to allow interpretation of the results. It is clearly a major undertaking, and an entirely separate piece of work to expand on this. I am not suggesting the authors do this, or that what they have done not is not worthy. However they absolutely need to acknowledge the limitations of data that they are

producing. The authors state their system extracts full (phenotype, rsID, pvalue) relations comparable to ones found in hand-curated databases, but neglect to mention the additional levels of richness of data that they do not extract.

The authors never mention GWAS study design, which makes me wonder if they even considered the meaning of the data they extract. I feel obliged to outline some basic points about GWAS analyses that they seem to have not considered.

There are two key metrics of a GWAS study. 1) the basic study design, 2) the power of the study.

Generally, a genome-wide analysis is performed (test of association of hundreds of thousands of SNPs distributed across the genome); this is the discovery stage. Often times, full genome-wide results of multiple cohorts are combined and meta-analysed. A problem with GWAS is the huge number of association tests that are run, and the associated likelihood of false positives. For this reason, the 'discovery' stage is very often followed by a 'replication' stage where a limited number of variants are examined in a second (or more) population. The combined, meta-analysed data are examined for statistical significance across all populations.

The power of a GWAS is determined largely by the number of individuals and the number of variants examined. Both of these metrics, at a minimum are required to interpret the results. Additionally, the ancestral background(s) of the studied population is increasingly acknowledged to be important. Knowledge of study power is invaluable when analysing data from a study. Assessment of whether results at a particular statistical significance level are real or due to chance requires knowledge of the study power.

All associations presented in Gwaskb are flat, presented without any information on study design and power, thus incredibly difficult (for the end user) to interpret.

My point here is not to speak to the quality of the data produced, or the machine reading system, but the authors do not seem to have considered the science of GWAS and the meaning of the data they wish to present. They do not give any space to acknowledging or discussing the interpretability and usability of the data.

They should, at minimum, note that the data they present may not actually be from a genome-wide analysis data. For example, they extract associations from replication stages even if it is not

significant at any other stage. Or even potentially from irrelevant statistical analyses (e.g. rsID with a p-value could be from non-association test).

Therefore the statement in the abstract ' our results demonstrate both the importance and the feasibility of automating the curation of the scientific literature' is a gross overstatement. As is 'we demonstrate that modern machine reading algorithms have matured to the point of significantly improving biomedical curation efforts' –This has not been demonstrated, particularly considering my next major point.

- Major point 2

My examination of the data presented indicates that the 'new' associations (found by GwasKB and not present in GWAS Catalog) are all associations that were deliberately excluded for scientific reasons. Not, as the authors claim, that they were 'missed' by human curators.

The authors do make the very valid claim (e.g. when discussing LD, lines 234-240) that all data should be available and not prefiltered, this is a point they should expand on with regard to different stages of analysis, different cohort etc. (see major point 1). However, as I can find no indication that any associations have been erroneously 'missed' they should reframe this discussion. They should keep in mind that the decisions on which SNPs should be excluded have been made for sound scientific and data standardisation reasons, and that they are aimed at generating particular type of database (one that contains high quality data).

The authors are not comparing like for like. They are effectively comparing two products that have been generated using completely different parameters and intentions and implying that the different results are not due to those underlying different parameters.

They claim to extract thousands of associations 'missed' by human curators, but they have neglected to examine the extraction guidelines by which the curators were working. I have examined a subset of the data presented (new associations found by GwasKB and 'missed' by human GWAS Catalog curators) and have not found a single example that could not explained by differences in extraction guidelines. I have annotated some associations in rels.discovered.annot.Rev_comments.xls (attached).

The main reasons data extracted was extracted by GwasKB and not extracted by GWAS Catalog were: 1) SNP not significant in all stages of analysis, 2) most significant SNP/locus excluded and other less significant SNPs not extracted.

These guidelines are in place for scientific reasons, to ensure consistent extraction of high quality data. The full methodology of the GWAS Catalog is available online (Methods section), and all of these extraction rules are outlined there. It seems remiss, even hubristic, of the authors not to have considered these before claiming 'human error' is the reason for the discrepancies.

I also found examples of associations, which the authors claim are missing, that ARE in the GWAS Catalog (PMID 23001564, rs1317082 (since 2012) ; PMID25064009, rs11724635 (since 2015)). This was a spot check of 15/100 associations, which does not encourage me about the quality of this analysis.

Please also supply column headers so that readers do not have to guess what the data are. What does the column with 0, 2 or 3 mean? In the annotation column there is reference to 'gold SNPs', what does this mean? There is also appears to be mix-ups in which comments refer to which SNPs (e.g. PMID19247474, rs1400363; PMID19300500, rs1877252).

- Major point 3

Throughout the paper it is very difficult to find the actual data to which the authors refer. Data that is specifically referred to should be included as Supplemental files. Having this data as Supp file will enable the authors to appropriately referred to it in the paper text.(E.g., but not limited to, associations.know.tsv, res.discovered.annotated.txt.). Currently this data is only available on github. Readers shouldn't have to download the entire repository to access results data (which is the case with github). It is unclear exactly what is to be found in the data files on github, and within the files there are no column headers. If some data is to remain on github alone, please provide a key, in the supplement, for all files provided on github with an explanation of their contents.

Generally, please support your claims by explicitly supplying and referring to your data.

Suggestion

At Line 79-82 authors state for extracted associations "We support our findings with evidence from publications, which can take the form of a sentence excerpt or a location in a table." This is also indicated in Figure 1. Where is this to be found? It doesn't appear in the 'results' files on github or the web interface. Please specify. If this data is currently not available it would be good to supply it.

If available, this feature could make Gwaskb a valuable tool for direct data users and curators. It could enable them to, in the former case go directly to the source for themselves to get a sense for the related study design/meta-data. It would be especially useful for manual curators as an aid to their work, allowing them to use their expertise at a high level and include or exclude associations based on their own requirements (extraction guidelines). Alternatively all associations could be annotated as appropriate with accurate meta-data (with onotology linked phenotype information, cohort, ancestral background of cohorts etc).

This function would be greatly improved, and practically probably contingent, on the expansion of Gwaskb to extracting additional data fields (OR/beta/allele etc). It would be also worth considering having the option of not having a p-value cut off (i.e. really extracting ALL data presented). For example if an association is significant in one cohort of many, it is valuable information that it was not significant the other cohorts.

Other points

- Pathway analysis.

Again, the authors have not actually supplied (that I can find) the data that would enable me to fully evaluate their claims. Please supply the actual data (variants, papers, traits).

The authors should consider that their Pathway Analysis of 'new associations' may be biased. The data analysed is associations with either neurodegenerative disease (ND) or autoimmune diseases (AD) that were not found in GWAS Catalog/Central. The variants are not in LD with known SNPs (from those publications in GWAS CAtalog/Central). These SNPs have largely (as far as I can tell) been excluded from the GWAS Catalog because they were not significant in all of the cohorts tested/are in same locus as a more significant SNP.

This data may be biased by what the authors of the original publications choose to show. For example, authors often choose to display results for SNPs at or near genes/loci that have been previously implicated in a related trait. They also selectively choose which SNPs to attempt for replication, based on prior knowledge about relevant loci/disease mechanism. This means they will often present 'suggestive' level significance data (p 1 x 10-5 to 10-8) for vaguely related loci, even for only one cohort/stage. Thus the prevalence of particular types of loci may be a kind of confirmation bias.

- Phenotypes

GWAS Catalog associations are labelled with two trait descriptors; one 'reported trait 'free text descriptor reflecting the authors language, as well as ontology terms (EFO). The authors don't mention which they use. I'm inferring they used the 'reported trait'.

The phenotypes supplied in GWASkb are 'candidates from EFO, Snomed and MeSH ontologies' (line 395). Can the authors expand on how these are integrated? How does the search interface work with respect to phenotypes.. is it ontology based? Allowing the data to be searched and synthesised across related traits is a key utility in association databases. Ideally, the data should be integrated using a common ontology. This is not absolutely necessary for this publication, but the authors should be clear about how the data is structured to allow users to search in an informed manner. (Also note that MeSH and Snomed are strictly not ontologies, they are vocabularies. They don't use the same strict hierarchical relationships used in ontologies.)

- Associations that GwasKB completely missed.

Line 192, 'in the remaining cases, we were not able to report the variant itself'. Please expand on the reasons why other associations were missed (apart from the very small number 89/147 that were not correct due to incorrect phenotype).

o Also provide 89/147 in percentage terms, as you have done for the correctly recovered associations. Not supplying this percentage, or the percentage of 'remaining cases' could look like you are trying to hide something.

- Effect size analysis

Figure 4 – to allow proper comparison please also add data from Fig S4 and Fig S3 (i.e. display GWASkb 'new' alongside GWAS Catalog effect sizes, as well as 'all'.)

It would be interesting to see a figure of effect sizes with 1) all GwasKB, all GWAS Cat, GWAS Cat NOT GwasKB, GwasKB NOT GWAS Cat, and GwasKB NOT GWAS Cat (LD pruned).

o Fig S4 (key is mislabelled as GwasKB, should be GWAS Cat.)

o Figure 4 – effect size of Gwas KB SNPs (LD pruned 'new' Gwas KB vs genome-wide SNPs) – please specify in the figure legend that these are LD pruned SNPs. Increase axis labelling to make it legible.

o Please list which LD hub studies were used for this analysis.

• Web interface:

o Associations are presented without a p-value if they are in LD with the lead SNP (I think). This information (SNP in LD with lead SNP) is useful, but this way of communicating it is very confusing and may mislead users. I suggest modifying this choice.

o The functionality is not robust. Searching for data that should be present in the database (e.g. 'new' associtions from 'rels.discovered_annot') often either produces an error ('stop iteration' screen) or a results page with no results. Occasional hanging on search and proxy errors. (I have noted some of these in the comments on rels.disc.annot_Rev_comments.xls.)

• This analysis was limited to publications whose full text is publically available, which I appreciate is a common constraint.. Could the authors expand on how in practice a user could use this tool on non-publically available texts?

• Inconsistency in name of tool/website. GwasKB, Gwasdb (github), Gwas archive (website). Pick one and stick with it. Or do they all refer to distinct things?

• Specify what version download of the GWAS Catalog data was used (new data is frequently released). Perhaps the 589 papers are listed somewhere on github, please list them in a supplementary table.

- Line 182 “we also specify whether our reported phenotype is exact or approximant”- where is this specified?

- Lines 186-193, again where is this data?

- Line 208 – refer to where this data can be found.

- Data referred to in lines 186-193, please supply this data as supplementary files and refer explicitly to where the data can be found.

- Line 197 - 2959 is provided as number of associations that could not be mapped to GWAS Catalog or Central. Line 226, 3170 is provided as the number of ‘new’ associations. In the ‘associations.new.tsv’ file there are 3318 associations.

  Is this an error or is there a reason these numbers are different?

- Line 231 – ‘over 40% of our discovered variants’, again specifically refer to the data, provided in a supplementary file. I don’t think this data (a list of the ‘new’ variants, post LD filtering) is provided. (If it were it would have saved this reviewer a lot of time going through variants from ‘rels.annot’ and finding that most of them were at the same locus as more significant variants i.e. deliberately excluded from the GWAS Catalog.)

- Lines – 234-240, this is a very valid point. There are strong arguments for presenting all data without LD filtering.

- Line 251 – strange syntax (‘we collected’)

- Supplemental materials, pathway analysis.

o Clarify the explanation of how this analysis was performed. Supply the actual data, including the variants and the genes.

o Change contingency table header from ‘Found in xx genes’ to ‘Found within 200kb of xx genes’ or ‘Found near xx genes’

• Cross-disease analysis;

o Again present both Fig 5 and Fig S5 together to allow comparison.

o This analysis seems unnecessary. Please provide more rationale for why this analysis was performed, in particular address the circularity (diseases that are known to be linked were chosen, then the conclusion is those diseases are linked?).

o The final example association (rs6857) is reported by the authors as a ‘new’ association with Alzheimer’s and LDL cholesterol. The actual phenotype studied was response to statin therapy, which is qualitatively a different trait to baseline LDL cholesterol levels. (This association not included in GWAS Catalog as it was not replicated.)

• Line 430 ‘Mapping Phenotypes across databases’. The authors provide tables with GWASkb traits mapped to GWAS Cat/Central. Again, please provide as supp data and refer specifically. I think the relevant files are phenotype.mapping.gwascat.annotated and phenotype.mapping.annotated. I don’t know what is in the different columns and I assume the column with the digits 0, 2 or 3 has something to do with the assessment of how close the mapping is (fully or partially correct). I’m basing my assessment of this data on guessing what is in the columns.

• On the web interface (and perhaps elsewhere) variants are referred to as ‘mutations’. The vast majority of variants examined in GWAS are common variants present in at least 5% of the population). It is completely inappropriate it refer to these as ‘mutations’. Also note that there are many traits that are not ‘diseases’ in GWASkb.

• Typos in Supp methods (LD), PLINK should be capitalised

• Line 35 – “absent in all others”, only two data sources were examined.

The authors should bear in mind some key differences between the GWAS Catalog and GWAS Central.

Note that not all GWAS Catalog and GWAS Central data is independently extracted. GWAS Central imports all GWAS Catalog data (GWAS Catalog does not import GWAS Central data). GWAS Central, I believe, does not do manual curation on all papers, in some cases the only data available is that taken from the GWAS Catalog.

GWAS Central also accepts user-submitted data, which has not necessarily appeared in the publication. Therefore there could be associations in GWAS Central which is not accessible to the authors' search algorithm.

A Machine-Compiled Database of Genome-Wide Association Studies

Response to reviewers

Editor response

We thank the editor and the reviewers for their detailed feedback on our paper. In this document, we provide a point-by-point response to the reviewers' comments.

- To address the first major concern of Reviewer 3, we have significantly rephrased the claims made in our paper, and we have included a more detailed analysis of the limitations of our method.
- To address the second major concern of Reviewer 3, we have performed an updated analysis of 100 random associations not present in the GWAS Catalog but found by our system. Our initial set of random associations contained variants that were in LD with variants from the GWAS Catalog (hence were omitted for scientific reasons). We have filtered these variants and have provided a detailed explanation of why the variants in our latest dataset were omitted.
- To address the third major concern of Reviewer 3, we have added a detailed explanation of the data and the results that are made available alongside our manuscript. We have also corrected the noted anomalies in our manuscript, public Github repository, and our web interface.
- Finally, in response to concerns raised by multiple reviewers, we have included an additional description of our novel statistical modeling method, data programming. We also corrected multiple typos, inconsistencies, and other minor issues that were brought to our attention.

As part of our response, we are also releasing several new materials, which we include both in our Github repository, as well as in the Supplementary Files:

- The full set of (variant, p-values) tuples extracted by our system, including ones that are not significant. Releasing this file has been suggested by reviewers 2 and 3, as it could help curators to identify and validate new significant variants more quickly.
- Additional metadata making it easier for users to evaluate the significance of the variants produced by our system, GWASkb. This metadata makes it easier to identify the stage of the GWAS study associated with each p-value.
- Additional notebooks that can be used to fully reproduce our biological analysis.
- All the data used by our system, including secondary ontologies and other data files

As requested by Reviewer 3, we are attaching to our submission a set of Supplementary Files, which includes all the materials present in the github repository.

We address the reviewers' questions and concerns in detail below.

Reviewer 1

We thank Reviewer 1 for their positive feedback; we address their comments below. Reviewer remarks are included in bold italicized font, in full.

The authors present a novel, data-programming approach, to machine-curation of GWAS associations. The results presented highlight that while such an approach should not totally replace the manual curation carried out by other resources, it has huge potential to lighten their workload and increase coverage. The authors themselves indeed suggest this joint approach in their well-balanced discussion.

The first assessment of their approach is the ability to recall previous, manually curated, associations from a set of 589 papers. Impressive performance is reported, with 69% of the associations from the GWAS catalog recalled with full accuracy in terms of the variant and phenotype, and 81% if allowing for approximately correct phenotypes. However, much of the workload of the manual curators in the GWAS catalog revolves around consistently and precisely defining the phenotypes using ontologies so that identical and/or related studies can easily be identified. The example in Table 2 of a fully accurate match with the GWAS catalog obviously describes the same phenotype but with a different text string: "Clozapine – Triglycerides". Would other association describing this same phenotype be labelled with different text strings using their approach, making it harder to identify identical studies for meta-analysis?

> We agree with the reviewer than not linking GWASkb phenotypes with existing phenotypes ontologies is a limitation of this approach.
>
> Mapping specific text *mentions* of phenotypes to their canonical identifiers in an ontology is a task known as *entity linking*, and is a non-trivial but standard component of many information extraction pipelines. Thus, while outside the scope of the present work, the data we have released is enough to perform a follow-up study that would produce such a mapping.

Given the missed 19% manually curated associations are likely to be high quality and of interest to researchers, it would seem manual curation is still beneficial. It will be interesting to discuss how much time having the 81% already identified will save?

> Our dataset contains about 80% of the associations that have been compiled in human-curated databases. However, any automatically extracted variant should also be manually validated. Assuming conservatively that validating variants takes 50% of the time that it takes to find those same variants in a large corpus of studies, we estimate that a machine-aided approach like ours would have saved about 40% of the total time required to compile a database like GWAS Central.
>
> As suggested by reviewer 3, we are also releasing the full set of (variant, p-values) tuples extracted by our system, including ones that are not significant. This larger dataset can be used by curators to prioritize their curation efforts within a moderately sized corpus of variants with a very high recall and a reasonably high precision. This data is included in our Supplementary Files:

- **Variants and p-values**
  - This file contains the variants (identified by their rsid) extracted by our system and every p-value that was found for them. Specifically, each row contains the paper pmid, variant rsid, table number, row number, column number that indicate where the rsid was found, and finally the log10(p-value). The p-value is always found in the same row as the rsid.
  - https://github.com/kuleshov/gwaskb/blob/master/notebooks/results/nb-output/pval-rsid.filtered.tsv
- **p-value Metadata**

- This file contains meta-data extracted for each (rsid, p-value) tuple that we report. Note that the format of this file is the same as that of the pval-rsid file.
- https://github.com/kuleshov/gwaskb/blob/master/notebooks/results/metadata/pval-rsid.metadata.tsv

The more impressive result is the additional 2959 relations discovered in the set of papers that had already been curated for the GWAS catalog. Strong functional evidence is presented for many being accurate and worthy of inclusion in a GWAS database and having the results of GwasKB available for manual curators would clearly be beneficial. The authors have made great efforts to make the code and data openly accessible for such a purpose. The manual inspection of 100 randomly chosen examples suggested 60 should pass the criteria of the manual curators of resources such as GWAS catalog. Assuming this is the case, and extends more widely, it would seem an extraordinary miss-rate by the manual curation approach. Can the authors comment on why they think so many were missed by manual curation? Are the majority in LD with one of the manually curated variants from the same study and rejected by the manual curators as suggested in the subsequent section? Or is it simple, human error as suggested in the discussion. This is key to others assessing whether to incorporate GwasKB as part of their curation pipeline.

At a high level, many of the extra annotations that our approach found can be interpreted as correctly excluded from GWAS Catalog according to their specific annotation guidelines, but nonetheless might be useful to researchers. We emphasize that a broad advantage of our approach is that it is far more flexible than a manual annotation approach. Rather than having to re-annotate a corpus given a change in annotation guidelines, we can simply edit a minimal amount of code.

Concretely, there are several reasons for extractions found by our approach but not present in GWAS Catalog, and we edited our paper to be maximally transparent about the reason why we observe these novel variants. We also conducted additional experiments (with a random sample of 100 associations not present in the GWAS Catalog but found by GWASkb) and revised our estimates. The main reasons for observing new variants as compared to GWAS Catalog are:

- The variants are in the same LD locus as other variants (35/100), and are either in weak LD (26/100), or strong LD (9/100). GWAS Catalog (the main database against which we compared) only chooses the most significant variant in each locus. However, our results show that the same locus can harbor variants that are only in weak LD with each other (e.g. $r^2<0.5$); therefore, it is valuable to report such variants. Furthermore, the LD cutoff may change later, and it is preferable to report all variants in a locus, and let the user perform their own filtering.
- The variants are not significant in every cohort studied in the paper (43/100). GWAS Catalog only includes variants that are significant at 10^{-5} in the meta-analysis (joint discovery and replication) cohort. We believe that there are cases when a larger set of variants may be useful (e.g. when testing for enrichment of variants in specific genomic regions), and in those cases, it is advisable to include variants that have been significant in any cohort. Our system reports all such variants, and in order to make it easier for users to filter them, we have compiled a list of meta-data that provides an indication of which cohort was used to determine an association. Once again, we emphasize that our automated approach is intended not just to extract relations according to the specific GWAS Catalog schema considered, but more generally to support customizable schemas.
- Some variants have been found to be significant in other papers (12/100), the given publication reproduces earlier results, and is either significant in the current paper (7/100) or not significant in the current paper (5/100). Oftentimes, the given study also reports such variants to be significant, and in that case, it makes sense to include them.

- Finally, we did observe a small number of cases (3/100) when a variant appears to have passed all the inclusion criteria of GWAS Catalog, but was not included.
- The remaining variants (7/100) are extractions errors made by GWASkb, reflecting the fact that its precision, while high, is not perfect.

For further details and additional discussion, please see our response to Reviewer 3 about this analysis.

Reviewer 2

We thank Reviewer 2 for their positive feedback; we address their main comments below. Reviewer remarks are included in bold italicized font, in full.

This is a useful project and well written manuscript. The authors address an important issue, viz, the fact that not all GWAS hits reported in manuscripts make it into databases such as GWAS Catalog, which are widely used for many downstream analyses. Thus, the community is not taking full advantage of published data because of the difficulties in curating and integrating this data. They propose a machine learning system based on the novel data-programming paradigm. The results are available on a website. For me, the most interesting part of the manuscript was the demonstration of the utility of the data programming paradigm for a medical data mining challenge. My main suggestion for the authors is that they should strive to provide a more detailed description and motivation of this methodology.

We have provided a more substantive high-level description of the data programming methodology in the main body of the paper, along with additional details on the overall technical approach taken. In the Supplementary Materials we have added a substantial (2 page) section on data programming, providing background details of the approach---including precise definitions of the model and learning algorithm used. And in the Supplementary Files we have included a file containing all of the labeling functions used for data programming:

- https://github.com/kuleshov/gwaskb/blob/master/notebooks/lfs.py

They also do not really compare their work against previous work, and they should provide more of a discussion/comparison.

Extracting structured relations from unstructured text is the subject of the field of information extraction (IE); our work is part of this field. We use a new approach to IE called data programming, and we are one of the first papers to apply it on a large scale.

Information extraction is widely used in diverse domains such as news, finance, geology, and in the biomedical domain. In the biomedical setting, IE systems have been used to parse electronic medical records, identify drug-drug interactions, and associate genotypes with drug response.

A considerable amount of effort has gone into uncovering gene/disease associations from biomedical literature. Our approach, however, takes a different approach, as it attempts to identify the effects of individual mutations.

Recently, Jain et al. applied information extraction to the GWAS domain; their work focused on creating extractors for two specific relations: paper phenotypes and subject ethnicities; these extractors achieved an 87% precision-at-2 and a 83% F1-score on the two tasks, respectively. In contrast, our works introduces an end-to-end system that extracts full (phenotype, rsid, pvalue) relations comparable to ones found in hand-curated databases.

These are the citations that are relevant for this work. We include all of them in our main paper.

- Moens, M. Information Extraction: Algorithms and Prospects in a Retrieval Context. (Springer Netherlands: 2009).
- Tumarkin, R. & Whitelaw, R.F. News or Noise? Internet Postings and Stock Prices. Financial Analysts Journal 57, 41-51 (2001).
- Das, S. & Chen, M. Yahoo! for Amazon: Extracting Market Sentiment from Stock Message Boards. Proceedings of the Asia Pacific Finance Association Annual Conference (APFA) (2001).

- Zhang, C. et al. GeoDeepDive: Statistical Inference Using Familiar Data-processing Languages. Proceedings of the 2013 ACM SIGMOD International Conference on Management of Data 993-996 (2013).doi:10.1145/2463676.2463680
- Zhou, X., Han, H., Chankai, I., Prestrud, A. & Brooks, A. Approaches to Text Mining for Clinical Medical Records. Proceedings of the 2006 ACM Symposium on Applied Computing 235-239 (2006).doi:10.1145/1141277.1141330
- Percha, B., Garten, Y. & Altman, R.B. Discovery and explanation of drug-drug interactions via text mining. Pacific Symposium on Biocomputing. Pacific Symposium on Biocomputing 410-421 (2012).
- Rinaldi, F., Schneider, G. & Clematide, S. Relation Mining Experiments in the Pharmacogenomics Domain. J. of Biomedical Informatics 45, 851-861 (2012).
- Pletscher-Frankild, S., Pallej a, A., Tsafou, K., Binder, J.X. & Jensen, L.J. DISEASES: Text mining and data integration of disease,Ägene associations. bioRxiv 008425+ (2014).doi:10.1101/008425
- Jain, S. et al. Weakly supervised learning of biomedical information extraction from curated data. BMC Bioinformatics 17, S1 (2016).

Minor comments.
1) The number of these new variants corresponds to about 20% of all open access associations recorded in the most up-to-date human-curated database, GWAS Catalog.
=> Is it true that GWAS catalog is the most up to date? What about GWASdb anbd GWAS Central etc.? Can the authors substantiate this claim? Or is the claim just based on the open access papers they examine?

Yes, our claim is mainly based on the open access papers that we examine. The relative completeness of the databases is somewhat more nuanced than we stated in our original draft, and we now provide clarifications here and in the Supplementary Materials of our paper.

The GWAS Central database contains fewer associations than GWAS Catalog among open-access papers. We have compiled a table describing these statistics:

**Database**	Statistics over open-access papers		
	**Papers**	**Associations**	**Unique Associations**
**GWAS Catalog**	589	8,384	>2,026
**GWAS Central**	516	5,914	>364
**GWASkb (ours)**	589	6,231	>2,777

The reason for this difference appears to be because GWAS Central has only been publishing self-reported associations (from authors) since 2015. Thus, several more recent studies that were not reported directly by researchers are not present. We also looked at GwasDB, but it appears to have not been updated since 2015.

On the other hand, GWAS Central contains a significantly larger number of associations. Instead of only considering associations that are significant at 1E-05 in the meta-analysis cohort, GWAS Central reports all the findings in the paper without any filtering. In addition, it allows authors to directly submit associations to the database. Thus, although it contains fewer recent open-access studies, it contains a more diverse set of associations.

Overall, we found that GWAS Catalog appeared to be the most actively maintained, had more transparent and well-defined inclusion rules, had a more convenient interface, and has the most easily downloadable contents. Consequently, we assume that researchers would be most likely to use the GWAS Catalog in their work. For these reasons (and because of its greater

completeness across more recent open-access studies), we compare more thoroughly to GWAS Catalog, although we report many statistics relative to GWAS Central as well.

We have added this discussion to the Supplementary Materials of our paper, and are have corrected the relevant paragraphs of the main paper. We are referring to the GWAS Catalog as “a popular human-curated database” rather than “the most complete”.

2) With GwasKB, we restricted ourselves to open-access papers,
=> Have the authors tried to text-mine non-open access papers? Surely they have access to many in their university.

We have not tried to mine non-open access papers. Mining non-open access papers currently represents a legal gray area, with certain publishers restricting the access to their papers for text mining. The rights given to readers greatly differ across publishers, and identifying publishers that allow text mining imposes a significant overhead when developing and deploying our system. Although we most likely have the right to mine non-open access papers internally, we may not be allowed to release this data or report our results. However, our system is designed to take arbitrary XML documents as input, and it would be easy to adapt it to run over any paper in this format that a user has access to.

3) Finally, in the classification stage, we determine which of these candidates are actually correct relation mentions using a 135 machine learning classifier.
=> It would be better to say "...we predict..." rather than " we determine"

We thank the reviewer for this edit, and have implemented it.

4) Finally, in the classification stage,we determine which of these candidates are actually correct relation mentions using a machine learning classifier. We use a Naive Bayes classifier with a small number of hand-crafted features (between 4 and 12) => Did the authors tune the classifier for each type of phenotype? How did they do so and what were the criteria? They should provide more detail here. Also, they should provide more detail as to how the best candidate features were chosen. I think that an example would be helpful, and the methodology should be described so that in principle it could be reproduced based on the description in the methods section (I realize that much code is available as Python notebooks, which is excellent, but I still think that the methods section needs to have a more precise overview of the methods).

We did not tune the classifier for each phenotype. We use a single classifier that takes as input substrings from the paper’s title and abstract and outputs a probability that this substring describes a phenotype studied in the paper. The other tasks (e.g. identifying the detailed phenotype, or identifying acronyms) use a different classifier. We use one classifier for each relation that we extract, and there are five such relations (as described in the figure)

The features were chosen based on a training/development set of 100 papers. These include an indicator for whether the paper is in the abstract or in the title, the word count of the sentence, the number of times the substring is repeated in the text, etc. The full list of features targeted are included in the Supplementary Files:

- https://github.com/kuleshov/gwaskb/blob/master/notebooks/lfs.py

5) line 260 We should note however that the vast majority of ND and AU variants were found far from coding regions => I think you mean AI variants (not AU)

We thank the reviewer for this edit, and have implemented it.

6) Examining the Effect Sizes of Novel GwasKB Variants => It is unclear to me why the authors perform this comparison since the estimated effect size is related to the p value cutoff they use and so their finding is an expected one.

> We agree with this reviewer that p-values are intimately linked to the effect size. However, because the effect sizes were generally obtained from another GWAS study, and not the study from which this SNP was extract from, this acts as an additional replication. SNPs identified using our approach are associated to the trait in multiple different cohorts and therefore are expected to be replicable.

7) In addition, past studies still need to be curated. An ideal solution appears to involve a combination of authors, machines, and curators => It would be great if the authors would contact the maintainers of one of the current GWAS databases and agree to collaborate (Note: this reviewer is not connected with any such database). Presumably the results of the authors' method could be used to improve the quality and scope of manual curation, thus providing the greatest utility to the community.

> In addition to open-sourcing our code and underlying methods, we have indeed reached out to the maintainers, and are hopeful that this will lead to a collaboration as suggested.

Reviewer 3

We thank the reviewer for their thorough analysis of our work. In light of their suggestions, we have edited the paper in order to provide a fair description of our contributions and the limitations of our method. In addition, we have also performed several new experiments.

We address the reviewer's specific concerns below. Reviewer remarks are included in bold italicized font, in full, and in italics only when repeated for an out-of-order response:

Kuleshov et.al. have created a machine learning algorithm that extracts genetic associations from the literature. The associations are in the form of variant identifier (rsID), statistic of association (p-value) and the phenotype that the variant is associated (publication wide, high-level; and detailed). The authors test this extraction system on a set of open-access publications that have been included in the GWAS Catalog, a manually curated database of associations. They also do some validation using data from GWAS Central. The authors claim to extract 80% of known associations (those already in GWAS Cat/Central) and an additional approx three to four thousand associations not present in GWAS Cat/Central. The authors perform some analysis (pathway analysis, effect size analysis) on the 'new' associations to assert their functional relevance.

I should state at the outse that this reviewer is associated with the GWAS Catalog. Although this review will sound largely negative I want to emphasise that I am broadly positive about this work and view it is incredibly important and potentially very useful. I see the future of curation of this type of data as a collaboration between man and machine (which the authors also seem to). GWAS is particularly suited to machine extraction as the basic unit of the data is fairly stereotyped. Therefore this effort should be encouraged. I am very keen on seeing machine learning used to advance the public availability of data.

However, the paper broadly reads like it is written by machine learning computationalists who have not fully considered the underlying biology or the requirements of the downstream data consumer, who have not sufficiently investigated their reference data/truth set and who are overstating the utility of their product. If they re-write the paper, bearing in mind the points I outline below, GwasKB could constitute a step towards a very useful resource.

1. ***Major point 1***
Extraction of three pieces of information (rsID, p-value, phenotype) is minimal. There are many other data types the authors could extract, which they do mention they could expand to (e.g. effect size-beta/OR, SE, allele). Extraction of these would greatly enhance the ultility of the data, but I see this as a natural extension of the work and I'm not concerned at present that they are not extracted yet. What I am concerned with, given how the authors present their data, is the lack meta-data provided to allow interpretation of the results. It is clearly a major undertaking, and an entirely separate piece of work to expand on this. I am not suggesting the authors do this, or that what they have done not is not worthy. However they absolutely need to acknowledge the limitations of data that they are producing. The authors state their system extracts full (phenotype, rsID, pvalue) relations comparable to ones found in hand-curated databases, but neglect to mention the additional levels of richness of data that they do not extract.

The authors never mention GWAS study design, which makes me wonder if they even considered the meaning of the data they extract. I feel obliged to outline some basic points about GWAS analyses that they seem to have not considered.

There are two key metrics of a GWAS study. 1) the basic study design, 2) the power of the study.

Generally, a genome-wide analysis is performed (test of association of hundreds of thousands of SNPs distributed across the genome); this is the discovery stage. Often times, full genome-wide results of multiple cohorts are combined and meta-analysed. A problem with GWAS is the huge

number of association tests that are run, and the associated likelihood of false positives. For this reason, the 'discovery' stage is very often followed by a 'replication' stage where a limited number of variants are examined in a second (or more) population. The combined, meta-analysed data are examined for statistical significance across all populations.

The power of a GWAS is determined largely by the number of individuals and the number of variants examined. Both of these metrics, at a minimum are required to interpret the results. Additionally, the ancestral background(s) of the studied population is increasingly acknowledged to be important. Knowledge of study power is invaluable when analysing data from a study. Assessment of whether results at a particular statistical significance level are real or due to chance requires knowledge of the study power.

All associations presented in Gwaskb are flat, presented without any information on study design and power, thus incredibly difficult (for the end user) to interpret.

My point here is not to speak to the quality of the data produced, or the machine reading system, but the authors do not seem to have considered the science of GWAS and the meaning of the data they wish to present. They do not give any space to acknowledging or discussing the interpretability and usability of the data.

In order to better emphasize the limitations of our approach, we have added the following additional material to the paper.

1.1. (GWAS study design) *The authors never mention GWAS study design. All associations presented in Gwaskb are flat, presented without any information on study design and power, thus incredibly difficult (for the end user) to interpret.*

We have added the following description of the design of a GWAS study. We point out the distinction between discovery and replication stages, and we discuss study power.

> Genome-wide associations are typically identified in a discovery cohort and then replicated in a separate replication cohort. Some curation projects (such as GWAS Catalog) only include associations that have been successfully replicated, while others (such GWAS Central) tend to include most associations. GWASkb follows the latter approach; this offers more flexibility and allows researchers to refine the data according to the level of confidence that best suits their needs.
>
> [In Supplementary Materials:] Genome-wide association studies (GWAS) are widely used for measuring the effects of genetic mutations on human traits. These are typically large case-control studies in which hundreds of thousands or more variants are measured in each study participant. The variants that are significantly enriched in one cohort versus the other are reported.
>
> In order to reduce the frequency of false positives, GWAS often consist of a discovery stage that is then followed by one or more replication stages. The final results are obtained from a meta-analysis of all the cohorts. The power of a GWAS is a function of both the number of variants and the number of participants in the study. In addition, other information, such as the ethnicity of the study or the statistical methodology used are important for understanding the results of a GWAS.
>
> Our work focuses on extracting triples of (variant, phenotype, p-value). A notable limitation of our system is that it does not output study sizes or populations. In the current version of GWASkb, this information needs to be extracted manually; however, this only needs to be done once per paper (rather than for every association).

1.2. (Acknowledging limitations of extraction method). ***They should, at minimum, note that the data they present may not actually be from a genome-wide analysis data. For example, they extract associations from replication stages even if it is not significant at any other stage. Or even potentially from irrelevant statistical analyses (e.g. rsID with a p-value could be from non-association test).***

We have added several paragraphs describing the limitations of our method, in that it does not yet accurately distinguish between discovery and replication p-values:

> For the purpose of evaluating the precision and recall of our system, we formed a dataset of all automatically extracted associations that were determined to be significant at p < 10-5 in at least one experiment in the study (such as in one cohort or one statistical model). This criterion recovered a significant number of associations present in existing databases, while maintaining sufficiently high precision (Table 1).
>
> It is important to note that our inclusion criterion is different from the one used by databases such as the GWAS Catalog, which includes only associations that are significant in a combined discovery and replication cohort. Our criterion is most similar to that of GWAS Central, which accepts all associations. The latter approach is more flexible, as it allows researchers to refine the data according to their needs.
>
> A disadvantage of this approach is that it is also includes low-confidence associations, such as ones that have not been replicated, that originate from an earlier study, or that may arise from non-GWA experiments. A lower-confidence dataset may still be useful for certain applications, such as for testing whether certain pathways are enriched for associated variants. However, this also requires manually filtering variants that do not meet the significance threshold for other applications, which can be burdensome.
>
> To assist with this process, we are releasing metadata that helps identify the cohort associated with a given variant (see Online Methods). This metadata can later be used to train classifiers that automatically identify the target cohort.

1.3. (Lack of metadata to interpret results) [Repeated from above]: *What I am concerned with, given how the authors present their data, is the lack meta-data provided to allow interpretation of the results. It is clearly a major undertaking […] However they absolutely need to acknowledge the limitations of data that they are producing.*

In addition to acknowledging the limitations of our results, we are including additional meta-data that makes it easier for users to evaluate the significance of the variants produced by GWASkb.

> One of the main limitations of the current version of GWASkb is that it cannot determine in a fully automated way the cohort or the methodology used to identify an association. For example, it does not automatically report whether a particular p-value is from a discovery cohort, or from a meta-analysis, or from one of three ethnicities studied in the paper. In order to make it easier for users to obtain this information, we are extracting additional meta-data for each GWASkb p-value and providing it together with our set of associations. Specifically, we are extracting and providing in a separate file the contents of the first three cells in rows hierarchically above each p-value.
>
> The following table illustrates the meta-data that we output. In this example, we report for each p-value (red) a string that describes its cohort (green).

Table 2. Discovery and follow-up genotyping results.

				Discovery		Follow-up		Combined			Annotation	
Chr	SNP	A1/A2	AF	Effect (se)	P-value	Effect (se)	P-value	Effect (se)	P-value	N	Location	Nearest Gene
2	rs17775170	A/G	0.27	−4.79E-02 (0.011)	5.36E-06	3.70E-03 (0.008)	6.218E-01	−1.38E-02 (0.006)	2.43E-02	7284	intronic	SLC9A2
2	rs2165179	A/G	0.33	−4.85E-02 (0.010)	1.47E-06	7.46E-03 (0.015)	6.280E-01	−3.17E-02 (0.008)	1.77E-04	7264	intronic	SCN3A

For example, the three p-values circled in red above would be associated with the following metadata (stored in a csv file):

doc_id	table_index	rows	cols	p-value	metadata
23056639	2	2	5	5.36e-06	[‘Discovery’, ‘P-value’]
23056639	2	2	7	6.218e-01	[‘Follow-up’, ‘P-value’]
23056639	2	2	9	2.43e-02	[‘Combined’, ‘P-value’]

More formally, this auxiliary meta-data is generated as follows. When a document is parsed, a Table object is created for each element in the document marked with <table> tags. A Table is composed of Cell objects that have a row start index, row end index, column start index, and column end index. Most Cells span only one column and run, but headers, for example, frequently span multiple rows, so we store row and column information in the more general format.

To find p-values in the tables, we use a regular expression that searches over the text in each cell. Where a p-value is found, we then iterate over the other cells in the table and save the text from any cell that overlaps with the column of the p-value, is in the top three rows of the table, and does not appear to be a p-value itself.

Ultimately, we want to add a component to GWASkb that will *predict* the cohort associated with the p-value. In the meantime, this auxiliary dataset will be helpful to users that want to manually look up in which study stages a given association was significant. Combined with gold standard GWAS Catalog data, this dataset can also be used in follow-up work to train machine learning classifiers for directly predicting the stage of a specific study.

The file we described is included in the Supplementary Files and the description we give here is included in an accompanying README in the same directory:

- https://github.com/kuleshov/gwaskb/blob/master/notebooks/results/metadata/pval-rsid.metadata.tsv

Finally, we are including the following additional pieces of metadata that will be useful for interpreting our results:

- Updated documentation for GWASkb, most notably a master list that documents all the output provided by our system:
  - https://github.com/kuleshov/gwaskb/blob/master/results.md
- An updated set of notebooks that reproduce the latest version of our results. These include notebooks for reproducing the biological analysis for the set of new variants (following the order of operations described in the above mentioned results.md):
  - https://github.com/kuleshov/gwaskb/tree/master/notebooks
- A new list of 100 randomly selected GWASkb variants that are not in the GWAS Catalog, as well as an analysis of why they were selected by our system:
  - https://github.com/kuleshov/gwaskb/blob/master/annotations/not_in_gwasc.xlsx

1.4. (Overstatement of claims) ***Therefore the statement in the abstract ' our results demonstrate both the importance and the feasibility of automating the curation of the scientific literature' is a gross overstatement. As is 'we demonstrate that modern machine reading algorithms have matured to the point of significantly improving biomedical curation efforts'–This has not been demonstrated, particularly considering my next major point.***

As suggested by the reviewer, we have removed our claim about having "demonstrated that machine reading algorithms have matured to the point of significantly improving biomedical curation efforts". We have also scaled back or removed other similar claims in the paper. The following table includes some examples.

**Before**	**After**
Our results demonstrate both the importance and the feasibility of automating the curation of scientific literature.	Our results represent a step towards using machine reading algorithms to help human curators synthesize knowledge in the biomedical literature.
As a result, independent human curation efforts are often not consistent, and even the largest GWAS databases are incomplete.	deleted
More generally, we demonstrate that modern machine reading algorithms have matured to the point of significantly improving biomedical curation efforts.	deleted
Despite revealing tens of thousands of genotype-phenotype associations, not all GWAS results are available to scientists in a structured form amenable to downstream analyses.	deleted

1.5. (Extraction of additional data types). [Repeated from above]: *Extraction of three pieces of information (rsID, p-value, phenotype) is minimal. There are many other data types the authors could extract, which they do mention they could expand to (e.g. effect size-beta/OR, SE, allele). Extraction of these would greatly enhance the ultility of the data, but I see this as a natural extension of the work and I'm not concerned at present that they are not extracted yet.*

We have added a paragraph that describes the data which is extracted by curators (cohort size, population, odds ratio, etc.) and emphasize that our system only considers three distinct data elements:

> At a high level, GWASkb, extracts genotype-phenotype relations from the biomedical literature and places them in a structured database (Figure 1). A typical association consists of a genetic variant, its associated phenotype, and a p-value indicating the significance of the association. GWASkb focuses on extracting these three characteristics. Associations also possess additional properties that our system does not yet process; these include an effect size, a risk allele, a target population, and others.

Major Point 2

My examination of the data presented indicates that the 'new' associations (found by GWASkb and not present in GWAS Catalog) are all associations that were deliberately excluded for scientific reasons. Not, as the authors claim, that they were 'missed' by human curators.

The authors do make the very valid claim (e.g. when discussing LD, lines 234-240) that all data should be available and not prefiltered, this is a point they should expand on with regard to different stages of analysis, different cohort etc. (see major point 1). However, as I can find no indication that any associations have been erroneously 'missed' they should reframe this discussion. They should keep in mind that the decisions on which SNPs should be excluded have been made for sound scientific and data standardisation reasons, and that they are aimed at

generating particular type of database (one that contains high quality data).

The authors are not comparing like for like. They are effectively comparing two products that have been generated using completely different parameters and intentions and implying that the different results are not due to those underlying different parameters.

They claim to extract thousands of associations 'missed' by human curators, but they have neglected to examine the extraction guidelines by which the curators were working. I have examined a subset of the data presented (new associations found by GwasKB and 'missed' by human GWAS Catalog curators) and have not found a single example that could not explained by differences in extraction guidelines. I have annotated some associations in rels.discovered.annot.Rev_comments.xls (attached).

The main reasons data extracted was extracted by GwasKB and not extracted by GWAS Catalog were: 1) SNP not significant in all stages of analysis, 2) most significant SNP/locus excluded and other less significant SNPs not extracted.

These guidelines are in place for scientific reasons, to ensure consistent extraction of high quality data. The full methodology of the GWAS Catalog is available online (Methods section), and all of these extraction rules are outlined there. It seems remiss, even hubristic, of the authors not to have considered these before claiming 'human error' is the reason for the discrepancies.

I also found examples of associations, which the authors claim are missing, that ARE in the GWAS Catalog (PMID 23001564, rs1317082 (since 2012) ; PMID25064009, rs11724635 (since 2015)). This was a spot check of 15/100 associations, which does not encourage me about the quality of this analysis.

Please also supply column headers so that readers do not have to guess what the data are. What does the column with 0, 2 or 3 mean? In the annotation column there is reference to 'gold SNPs', what does this mean? There is also appears to be mix-ups in which comments refer to which SNPs (e.g. PMID19247474, rs1400363; PMID19300500, rs1877252).

We agree that most of the 100 random novel variants included in our initial submission are in LD with GWAS Catalog variants, hence excluded for scientific reasons. This is due to an error on our part. We selected these variants before we filtered the output of our system to exclude variants that are in LD with GWAS Catalog variants. We then did not generate a new variant list after the filtering. However, we did use associations filtered for LD in all our other analyses, and this can be verified by looking at our Jupyter notebooks.

We have now collected and annotated a new set of 100 random associations not present in the GWAS Catalog and that have been pre-filtered based on LD. We have annotated each association with the following information:

- The reason for which the association is not in the GWAS Catalog.
- Whether the association was excluded due to curator error.
- Whether we recommend it for inclusion in a curated database. We base this recommendation on whether we think there are situations in which the association will be useful to researchers.

The 100 variants were not in the GWAS Catalog for one of the following reasons:

1. **[43 variants]** Variants that are significant in one analysis cohort, but not in the combined meta-analysis.
    a. Such associations are generally low-confidence. However, we believe that there are applications in which they may be useful (e.g. enrichment analyses). Therefore, we include them as part of output, but in order to make it easy to filter them out, we have extracted a set of meta-data for each

variant (and described above); this meta-data can be used by researchers to prune low-confidence associations that were not significant in the meta-analysis.

2. **[26 variants]** Variant is in the same locus as a more significant variant that is also in the GWAS Catalog. However, the LD between these two variants is weak.
    a. Even though two variants are in same locus (i.e. within the same genomic region) they may not be in strong LD. We found this happened quite often; we validated our estimated LD numbers (these were derived from the 1000 Genomes dataset) with an online tool from the NIH. In our analysis we used $r^2 < 0.5$ in the most precise population available for the study (e.g. CEU, EUR, ALL) as a threshold for what constitutes “weak LD”. When the LD is weak according to both our estimates and the NIH tool, we believe that cataloguing our proposed association would be useful to researchers.
3. **[9 variants]** Variant is in the same locus as a more significant variant that is in also in the GWAS Catalog. The LD between these two variants is strong.
    a. These variants may not be useful as the variants that are in weak LD. However, including them may be still useful in some uses cases, because the LD cutoff for what constitutes a strongly correlated variant may change in the future. Collecting these variants allows users to later select the subset of the data that is relevant to their needs.
4. **[7 variants]** Variant appears in previous paper, but is also found to be significant in this paper.
    a. The variant was found to be significant in an earlier study, and in the discovery stage of the current study, but not in its meta-analysis stage. The GWAS Catalog guidelines indicate that such variants should be included, but we found cases when they were not. This can be interpreted as a small curator error.
5. **[5 variants]** Variant appears in previous paper, but is not found to be significant in this paper.
    a. The variant was found to be significant in an earlier study, but not the discovery stage of the current study, hence it was correctly not included in the GWAS Catalog.
6. **[3 variants]** Curation error in the GWAS Catalog.
    a. We also found a small number of associations that were excluded from GWAS Catalog due to what we believe to be curator errors. These variants appear to have been truly significant and passed replication, but were not curated.
7. [**7 variants]** GWASkb extraction error.
    a. We extracted an incorrect phenotype for these variants.

Most of the above variants have been excluded from the GWAS Catalog for scientific reasons. However, we recommend a large number of these variants for inclusion in a broader curated database, because they are still relevant to researchers. These include 7 variants that have been replicated from a previous study, 26 variants that are in the same locus as a GWAS Catalog variant, but whose LD is weak, as well as 3 curator errors (36 variants in total). In addition, 43 variants that have not been replicated in a meta-analysis and 9 variants that are in LD with GWAS Catalog variants at $r^2 \geq 0.5$ (50 variants in total) may also be useful in a limited number of applications, as described above. The remaining 12 variants are not worth curating, and represent a GWASkb error.

More broadly, we emphasize that our goal in the proposed approach was to give researchers increased flexibility with respect to the particular annotation guidelines used. In our automated approach, if researchers wish to populate the database according to different annotation guidelines, this can be accomplished simply by changing a minimal amount of code and re-running our pipeline- rather than needing to manually re-annotate an entire corpus.

We have edited the paper to reflect these findings, including the following excerpts:

- Our inclusion criterion is less stringent than that of the GWAS Catalog, but would be comparable to that of some other human-curated databases, such as GWAS Central.
- Providing an extended set of associations --- a large part of which is valid and can be efficiently verified --- has the potential to assist curators. The additional variants not in the GWAS Catalog can still be useful for certain analysis, but researchers need to use their judgment before using them.
- A small number of associations appear to have been omitted due to curator error. Our system also produces a small number of errors. For this reason, we recommend that all automated extractions be validated, though we expect the validation process to be much faster than discovery.

Major Concern 3
Throughout the paper it is very difficult to find the actual data to which the authors refer. Data that is specifically referred to should be included as Supplemental files. Having this data as Supp file will enable the authors to appropriately referred to it in the paper text.(E.g., but not limited to, associations.know.tsv, res.discovered.annotated.txt.). Currently this data is only available on github. Readers shouldn't have to download the entire repository to access results data (which is the case with github).

We have significantly updated our paper to document our results.

3.1. (Explanation of data on Github) ***It is unclear exactly what is to be found in the data files on github, and within the files there are no column headers. If some data is to remain on github alone, please provide a key, in the supplement, for all files provided on github with an explanation of their contents.***

Generally, please support your claims by explicitly supplying and referring to your data.

We have released a master list documenting all the files used by GWASkb as well as the files that it generates. This list is available both on Github and in the Supplementary Files. It also contains instructions for how to interpret each file (including column names). The locations of all files in the the Supplementary Files is provided in the README.md file and their contents are described in results.md:

- https://github.com/kuleshov/gwaskb/blob/master/README.md
- https://github.com/kuleshov/gwaskb/blob/master/results.md

3.2. (Making data available as supplementary file). [Repeated from above]: *Data that is specifically referred to should be included as Supplemental files. Having this data as Supp file will enable the authors to appropriately referred to it in the paper text.(E.g., but not limited to, associations.know.tsv, res.discovered.annotated.txt.).*

We have created an archive containing all the files generated by our system that is available both on Github and in our Supplementary Files (as described above). We will determine with the editor what the best way is to upload and release this file with the publication if accepted.

3.3. (Indicating where in the papers various data elements were found) ***At Line 79-82 authors state for extracted associations "We support our findings with evidence from publications, which can take the form of a sentence excerpt or a location in a table." This is also indicated in Figure 1. Where is this to be found? It doesn't appear in the 'results' files on github or the web interface. Please specify. If this data is currently not available it would be good to supply it.***

Our system is implemented in a series of Jupyter notebooks, each running a module of the system. Each module is responsible for extracting one type of data element (e.g. p-values). These intermediary outputs are recorded in the nb-output folder of the Supplementary Files:

- https://github.com/kuleshov/gwaskb/tree/master/notebooks/results/nb-output

The final notebook collects these outputs to form our final list of associations.

In each row of the intermediary output files (e.g. for each p-value), we provide coordinates that point to a location in a paper where that data element (e.g. the p-value) was found.

The following files are the intermediary outputs; they also contain the coordinates of the extracted data elements.

- **Variants)**
  - File containing the p-values and the variants (identfied by their rsid) extracted by our system. Specifically, each row contains the paper pmid, variant rsid, table number, row number, column number that indicate where the rsid was found, and finally the log10(p-value). The p-value is always found in the same row as the rsid.
  - https://github.com/kuleshov/gwaskb/tree/master/notebooks/results/nb-output/pval-rsid.filtered.tsv
- **p-values**
  - File containing the p-values extracted by GWASkb and their location within publications (represented by a paper pubmed id, a table id, and a row, column coordinate).
  - https://github.com/kuleshov/gwaskb/tree/master/notebooks/results/nb-output/p-values.tsv
- **Precise phenotypes**
  - this file contains precise phenotypes for variants. The first three columns are the Pubmed ID of the paper, the RSID of the variant, the phenotype that we identified for that variant. The last three columns also include the table, row, and column numbers that indicate where the rsid was found.
  - https://github.com/kuleshov/gwaskb/tree/master/notebooks/results/nb-output/phen-rsid.table.rel.all.tsv

Finally, the simple phenotypes that we extract are associated with the paper as a whole, and hence are not associated with specific coordinate. However, by slightly modifying our simple phenotype extraction notebooks, it would be easy to output all the sentence where that phenotype string was found.

3.3. (Extracting all p-values without the cutoff) ***If available, this feature could make Gwaskb a valuable tool for direct data users and curators. It could enable them to, in the former case go directly to the source for themselves to get a sense for the related study design/meta-data. It would be especially useful for manual curators as an aid to their work, allowing them to use their expertise at a high level and include or exclude associations based on their own requirements (extraction guidelines). Alternatively all associations could be annotated as appropriate with accurate meta-data (with onotology linked phenotype information, cohort, ancestral background of cohorts etc). This function would be greatly improved, and practically probably contingent, on the expansion of Gwaskb to extracting additional data fields (OR/beta/allele etc). It would be also worth considering having the option of not having a p-value cut off (i.e. really extracting ALL data presented). For example if an association is significant in one cohort of many, it is valuable information that it was not significant the other cohorts.***

As suggested by the reviewer, we are releasing the full set of (variant, p-values) tuples extracted by our system. These p-values are also augmented with meta-data that enables us to identify their significance. For each association, researchers can query this dataset to identify all the p-

values that have been extracted for the target variant and use the metadata to determine whether it was significant in every cohort.

This data is currently available in our Github repository in the following files. We are also including them in our Supplementary Materials file.

- **variants and p-values**
  - This file contains the variants (identified by their rsid) extracted by our system and every p-value that was found for them. Specifically, each row contains the paper pmid, variant rsid, table number, row number, column number that indicate where the rsid was found, and finally the log10(p-value). The p-value is always found in the same row as the rsid.
  - https://github.com/kuleshov/gwaskb/blob/master/notebooks/results/nb-output/pval-rsid.filtered.tsv
- **p-value metadata**
  - This file contains meta-data extracted for each (rsid, p-value) tuple that we report. Note that the format of this file is the same as that of the pval-rsid file.
  - https://github.com/kuleshov/gwaskb/blob/master/notebooks/results/metadata/pval-rsid.metadata.tsv

In practice, to test an association, a researcher can query that association's pmid and rsid in the above list and identify all the p-values that were extracted for that (pmid and rsid). Then, they can use the metadata to determine the cohort to associated with each of the p-values.

In conclusion, these files serve two useful purposes:
- They form a large set of associations that can be verified by curators, thus accelerating their work.
- They enable one to further filter our dataset for downstream applications that require a more stringent significance threshold.

3.4. (Pathway analysis notebooks) ***Pathway analysis: Again, the authors have not actually supplied (that I can find) the data that would enable me to fully evaluate their claims. Please supply the actual data (variants, papers, traits).***

The following notebooks can be used to reproduce the biological analysis of the set of "novel" variants identified by GWASkb.

- Enrichment:
  - This notebook reproduces the enrichment analysis of GWASkb variants associated with either auto-immune or neuro-degenerative diseases.
  - https://github.com/kuleshov/gwaskb/blob/master/notebooks/bio-analysis/enrichment/enrichment.ipynb
- Effect sizes:
  - This notebook reproduces the analysis of the effect sizes of variants identified by GWASkb.
  - https://github.com/kuleshov/gwaskb/blob/master/notebooks/bio-analysis/effect-sizes/effect-sizes.ipynb

Other Concerns
The authors should consider that their Pathway Analysis of 'new associations' may be biased. The data analysed is associations with either neurodegenerative disease (ND) or autoimmune diseases (AD) that were not found in GWAS Catalog/Central. The variants are not in LD with known SNPs (from those publications in GWAS Catalog/Central). These SNPs have largely (as far

as I can tell) been excluded from the GWAS Catalog because they were not significant in all of the cohorts tested/are in same locus as a more significant SNP.

This data may be biased by what the authors of the original publications choose to show. For example, authors often choose to display results for SNPs at or near genes/loci that have been previously implicated in a related trait. They also selectively choose which SNPs to attempt for replication, based on prior knowledge about relevant loci/disease mechanism. This means they will often present 'suggestive' level significance data (p 1 x 10-5 to 10-8) for vaguely related loci, even for only one cohort/stage. Thus the prevalence of particular types of loci may be a kind of confirmation bias.

We have published on Github two additional notebooks that we used to generate the results for our biological analysis. We have also added to the notebook and to the supplementary material the list of studies that we used, as well as the list of variants we analyzed.

- https://github.com/kuleshov/gwaskb/blob/master/notebooks/bio-analysis/enrichment/enrichment.ipynb
- https://github.com/kuleshov/gwaskb/blob/master/notebooks/bio-analysis/effect-sizes/effect-sizes.ipynb

In addition, we also want to clarify that the variants we analyze are not in LD with GWAS Catalog SNPs, and hence were most likely not excluded because "they are in the same locus as a more significant SNP" (unless the locus contains variants not in LD with the given variant). Therefore, it is unlikely that this is a source of bias. However, we agree that the variants we test may not be significant in all cohorts. Note also that as described in a previous comment, our set of 100 random SNPs was sampled before we filtered for LD, and is therefore not affected by this problem.

Phenotypes: GWAS Catalog associations are labelled with two trait descriptors; one 'reported trait 'free text descriptor reflecting the authors language, as well as ontology terms (EFO). The authors don't mention which they use. I'm inferring they used the 'reported trait'.

We used the reported trait in our analysis. We have added a clarification to the paper:

> GWAS Central and GWAS Catalog contain respectively 3008 and 4023 accessible associations linked to the 589 open-access studies (see the Supplementary Materials for a comparison of the contents of these two databases). These associations are defined as tuples of PubMed ID, variant RSID, phenotype, and p-value for which the RSID is contained in the open-access XML content made available through PubMed Central. For GWAS Catalog we use the reported trait for our analysis rather than ontology terms (EFO).

The phenotypes supplied in GWASkb are 'candidates from EFO, Snomed and MeSH ontologies' (line 395). Can the authors expand on how these are integrated? How does the search interface work with respect to phenotypes.. is it ontology based? Allowing the data to be searched and synthesised across related traits is a key utility in association databases. Ideally, the data should be integrated using a common ontology. This is not absolutely necessary for this publication, but the authors should be clear about how the data is structured to allow users to search in an informed manner. (Also note that MeSH and Snomed are strictly not ontologies, they are vocabularies. They don't use the same strict hierarchical relationships used in ontologies.)

We used these vocabularies and ontologies as follows.

The process of identifying the phenotype associated with a paper has two stages. First, we generate a large set of candidate substrings in the title and abstract; the goal of this stage is to generate a large set of potential phenotypes that will contain the true one that we want to extract. At the second stage, we use a machine learning classifier trained using data programming to assign a probability of being correct to each candidate. Finally, we choose the three most likely candidates whose probability is higher than a threshold.

Although we use the EFO ontology (in addition to the Snomed, and MeSH vocabularies) to generate candidates, we did not attempt to trace the origins of the candidates back to the ontology. This is a significant undertaking that is outside the scope of the present paper.

Finally, the reviewer asks about how the search function in GWASkb works. It currently uses string matching to search for phenotype names that contain the user's query as a substring.

Associations that GwasKB completely missed: Line 192, 'in the remaining cases, we were not able to report the variant itself'. Please expand on the reasons why other associations were missed (apart from the very small number 89/147 that were not correct due to incorrect phenotype).

We have updated our paper with the requested explanation by the reviewer. It now reads as follows:

> The dataset reported by GWASkb contained 2487 (82%) associations from GWAS Central with approximately correct phenotypes, as well as 3245 (81%) associations from the GWAS Catalog. It also recovered 1890 (63%) associations from GWAS Central and 2762 (69%) associations from GWAS Catalog with full accuracy on the phenotype. Some associations were not correctly recovered because their reported phenotype was incorrect: 89 (3%) in GWAS Central and 147 (4%) in GWAS Catalog. In the remaining cases, we were not able to report the variant itself. The main causes of this are when the variants are expressed only in the text and not in tables, or when the format of the table is particularly difficult to parse (e.g., when multiple RSIDs and p-values are reported in the same row). Overall, GWASkb recovered 81-82% of manually curated associations at a level of quality that will be useful in many applications.

Also provide 89/147 in percentage terms, as you have done for the correctly recovered associations. Not supplying this percentage, or the percentage of 'remaining cases' could look like you are trying to hide something.

We thank the reviewer for their detailed feedback, and have corrected this.

Effect size analysis: Figure 4 – to allow proper comparison please also add data from Fig S4 and Fig S3 (i.e. display GWASkb 'new' alongside GWAS Catalog effect sizes, as well as 'all'.) It would be interesting to see a figure of effect sizes with 1) all GwasKB, all GWAS Cat, GWAS Cat NOT GwasKB, GwasKB NOT GWAS Cat, and GwasKB NOT GWAS Cat (LD pruned).

Fig S4 (key is mislabelled as GwasKB, should be GWAS Cat.)

Figure 4 – effect size of Gwas KB SNPs (LD pruned 'new' Gwas KB vs genome-wide SNPs) – please specify in the figure legend that these are LD pruned SNPs. Increase axis labelling to make it legible.

Please list which LD hub studies were used for this analysis.

We again thank the reviewer for their attention to detail and helpful feedback- we have addressed these issues in the new draft.

Web interface: Associations are presented without a p-value if they are in LD with the lead SNP (I think). This information (SNP in LD with lead SNP) is useful, but this way of communicating it is very confusing and may mislead users. I suggest modifying this choice.

We thank the reviewer for bringing this anomaly to our attention. This is not the intended functionality and was due to a malfunction in the system. We have corrected the bug and now display the set of p-values that we extracted for each variant. Below is a screenshot of an SNP that the reviewer specifically pointed out which is now handled correctly:

.

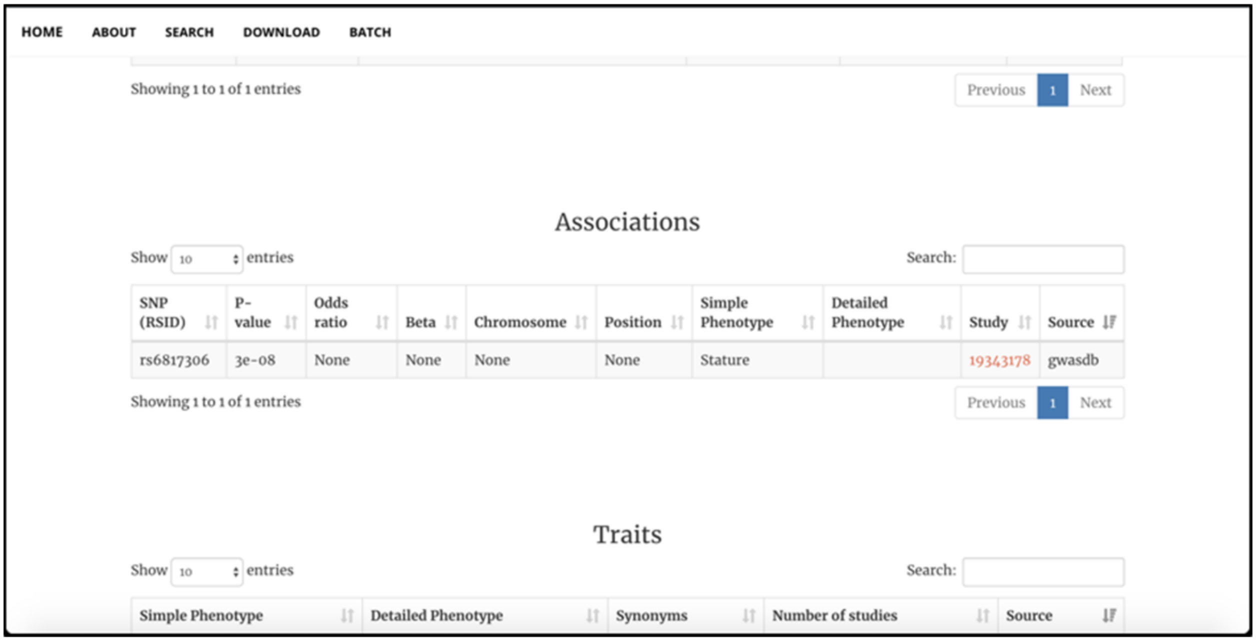

SNP (RSID)	P-value	Odds ratio	Beta	Chromosome	Position	Simple Phenotype	Detailed Phenotype	Study	Source
rs6817306	3e-08	None	None	None	None	Stature		19343178	gwasdb

The functionality is not robust. Searching for data that should be present in the database (e.g. ‘new’ associtions from ‘rels.discovered_annot’) often either produces an error (‘stop iteration’ screen) or a results page with no results. Occasional hanging on search and proxy errors. (I have noted some of these in the comments on rels.disc.annot_Rev_comments.xls.)

We have addressed these bugs and have verified the system. We have specifically double-checked the issues brought up in the Excel file. Shown below is one of the indicated searches that previously produced an error but now returns the proper results.

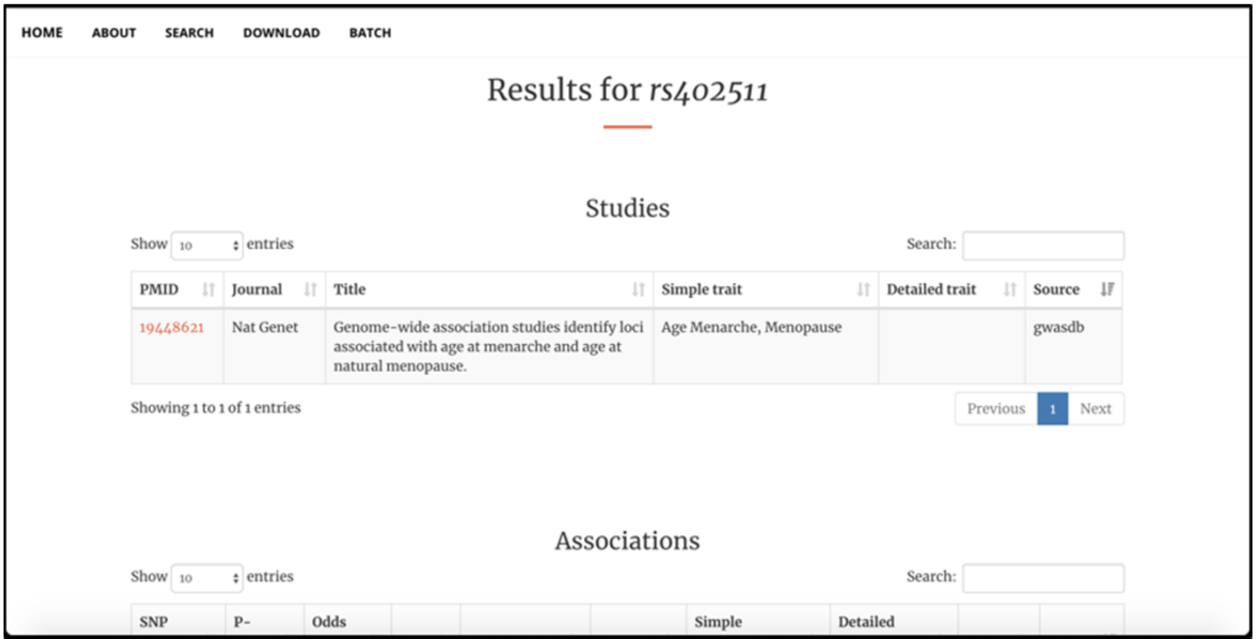

This analysis was limited to publications whose full text is publically available, which I appreciate is a common constraint.. Could the authors expand on how in practice a user could use this tool on non-publicly available texts?

The program accepts as inputs a folder with inputs files in XML format. The documents we used were part of a open-access paper release from PubMed. However, we can accept any paper in this format (as long as it uses standard HTML tables to format the GWAS results, and uses certain tags like <abstract> to denote specific parts of the paper). The best way to run the system on any paper is to go to the its Pubmed Central page, download the HTML source, and place it into the input paper folder.

Inconsistency in name of tool/website. GWASkb, Gwasdb (github), Gwas archive (website). Pick one and stick with it. Or do they all refer to distinct things?

We have corrected these inconsistencies to always use GWASkb.

Specify what version download of the GWAS Catalog data was used (new data is frequently released). Perhaps the 589 papers are listed somewhere on github, please list them in a supplementary table.

We have downloaded the contents of the GWAS Catalog on July 5, 2016.

Line 182 "we also specify whether our reported phenotype is exact or approximant"- where is this specified?

This is specified in the phenotype mapping table:

- https://github.com/kuleshov/gwaskb/blob/master/notebooks/util/phenotype.mapping.gwascat.annotated.tsv

Lines 186-193, again where is this data?

This data is references in our Jupyter Notebook #5 (notebooks/evaluation.ipynb), which walks the user step-by-step through how to obtain these numbers.

Line 208 – refer to where this data can be found.

This paragraphs refers to the 100-line Excel sheet of novel relations (that you annotated with your remarks), although we have now generated a new set of random relations (after filtering for LD), which can be found at:

- https://github.com/kuleshov/gwaskb/blob/master/annotations/not_in_gwasc.xlsx

Data referred to in lines 186-193, please supply this data as supplementary files and refer explicitly to where the data can be found.

We have included this in our master list:

- https://github.com/kuleshov/gwaskb/blob/master/results.md

Line 197 - 2959 is provided as number of associations that could not be mapped to GWAS Catalog or Central. Line 226, 3170 is provided as the number of 'new' associations. In the 'associations.new.tsv' file there are 3318 associations. Is this an error or is there a reason these numbers are different?

We thank the reviewer for pointing out this inconsistency. The correct numbers can be found in the analysis notebook in the most recent version of our results on Github:

- https://github.com/kuleshov/gwaskb/blob/master/notebooks/evaluation.ipynb

Manually evaluating precision

We measure precision over associations that haven't been confirmed by either GWAS Catalog or GWAS Central.

```
assocs_gwdb = set([(pmid, rsid, phen) for pmid, rsid, _, phen in associations])
print 'Relations in GwasKB: %d' % len(assocs_gwdb)

confirmed_assocs_gwcen, confirmed_assocs_gwcat = set(confirmed_assocs_gwcen), set(confirmed_assocs
_gwcat)
print 'Relations confirmed via GWAS Central: %d' % len(confirmed_assocs_gwcen)
print 'Relations confirmed via GWAS Catalog: %d' % len(confirmed_assocs_gwcat)

confirmed_assocs = (assocs_gwdb & set(confirmed_assocs_gwcen)) | (assocs_gwdb & set(confirmed_asso
cs_gwcat))
unconfirmed_assocs = assocs_gwdb - confirmed_assocs

print 'Relations confirmed via at least one database: %d' % len(confirmed_assocs)
print 'Unconfirmed assocs: %d' % len(unconfirmed_assocs)
```

```
Relations in GwasKB: 6376
Relations confirmed via GWAS Central: 2438
Relations confirmed via GWAS Catalog: 3235
Relations confirmed via at least one database: 3452
Unconfirmed assocs: 2924
```

Unfortunately, we re-ran our system with slightly different settings, and the numbers in the paper were no longer up to date. We have now updated our paper with the latest numbers.

Note that although there are 3318 entries in associations.new.tsv, some of these are correspond to repeated (paper, phenotype, variant) tuples with different p-value numbers (because the same variant can be found in multiple tables with different p-values in the same paper). Therefore the correct way to obtain the number of new associations is to filter for these repeats. We do that in the notebook, and the correct number is 2924.

Also, we report slightly different numbers in Table 1. These correspond to tuples of (paper, variant), as opposed to (paper, variant, phenotype). We omit the phenotype because of the difficulty of matching up equivalent phenotypes across databases. Note that reporting (paper, variant) tuples here gives an accurate picture of the size of each database, which is the main purpose of the table. Again, the code to produce these numbers lies in our evaluation notebook.

Line 231- 'Over 40% of our discovered variants', again specifically refer to the data, provided in a supplementary file. I don't think this data (a list of the 'new' variants, post LD filtering) is provided. (If it were it would have saved this reviewer a lot of time going through variants from 'rels.annot' and finding that most of them were at the same locus as more significant variants i.e. deliberately excluded from the GWAS Catalog.)

We used the 1000 Genomes panel to filter out novel variants that were in LD with known variants in the GWAS Catalog. We have included the output of this analysis in the following files:

- **Filtered associations**
  - We use the above LD data to filter associations that have R2 > 0.5 with some known GWAS Catalog variant. This uses the same format as associations.tsv, and we add one column, which is the LD to the strongest variant in GWAS Catalog.
  - https://github.com/kuleshov/gwaskb/blob/master/notebooks/bio-analysis/ld/associations.ld-filtered.tsv
- **Pairwise LD data**
  - Pairwise linkage desquilibrium between novel GWASkb variants and known GWAS Catalog variants. First column is the pmid of the variant, second column is the GWASkb variant, third column is the GWAS Catalog variant, and the fourth column is the LD (measured using the R2). For each pmid, we compute the pairwise LD from every GWASkb variant to every GWAS Catalog variant reported for that paper.
  - https://github.com/kuleshov/gwaskb/blob/master/notebooks/bio-analysis/ld/ld.mat

Also, these are the LD Link studies we used (they are available in the supplementary materials, in the notebook that performs this analysis):

Alzheimer's: http://web.pasteur-lille.fr/en/recherche/u744/igap/igap_download.php
Parkinson's: https://www.ncbi.nlm.nih.gov/projects/SNP/gViewer/gView.cgi?aid=2868
Schizophrenia, Autism, Smoking: https://www.med.unc.edu/pgc/results-and-downloads
Depression: https://www.thessgac.org/data
Type 2 Diabetes: http://diagram-consortium.org/downloads.html
Body Mass Index, Obesity:
http://portals.broadinstitute.org/collaboration/giant/index.php/GIANT_consortium_data_files
Asthma: http://www.cng.fr/gabriel/results.html
LDL Cholesterol: http://csg.sph.umich.edu//abecasis/public/lipids2013/

Lines – 234-240, this is a very valid point. There are strong arguments for presenting all data without LD filtering.

Line 251 – strange syntax ('we collected')

Supplemental materials, pathway analysis.

- Clarify the explanation of how this analysis was performed. Supply the actual data, including the variants and the genes.
- Change contingency table header from 'Found in xx genes' to 'Found within 200kb of xx genes' or 'Found near xx genes'

We have addressed these points.

Cross-disease analysis;
o Again present both Fig 5 and Fig S5 together to allow comparison.
o This analysis seems unnecessary. Please provide more rationale for why this analysis was performed, in particular address the circularity (diseases that are known to be linked were chosen, then the conclusion is those diseases are linked?).
o The final example association (rs6857) is reported by the authors as a 'new' association with Alzheimer's and LDL cholesterol. The actual phenotype studied was response to statin therapy, which is qualitatively a different trait to baseline LDL cholesterol levels. (This association not included in GWAS Catalog as it was not replicated.)

We thank the reviewer for this feedback and for pointing an error in this analysis. We agree that it overlaps in many ways with some of existing analyses, and for this reason we are removing it.

Line 430 'Mapping Phenotypes across databases'. The authors provide tables with GWASkb traits mapped to GWAS Cat/Central. Again, please provide as supp data and refer specifically. I think the relevant files are phenotype.mapping.gwascat.annotated and phenotype.mapping.annotated. I don't know what is in the different columns and I assume the column with the digits 0, 2 or 3 has something to do with the assessment of how close the mapping is (fully or partially correct). I'm basing my assessment of this data on guessing what is in the columns.

We have linked to these files in the master list:

- Mapping of GWAS Central phenotypes to GWASkb phenotypes. The columns are: GWAS Central phenotype, simple GWASkb phenotype, detailed GWASkb phenotype, code. The code is: 0=incorrect phenotype, 1=incorrect because acronym was not resolved, 2=approximately correct, 3=fully correct.
    - o https://github.com/kuleshov/gwaskb/blob/master/notebooks/results/util/phenotype.mapping.annotated.tsv
- Mapping of GWAS Catalog phenotypes to GWASkb phenotypes. The columns are: GWAS Catalog phenotype, simple GWASkb phenotype, detailed GWASkb phenotype, code. The code is: 0=incorrect phenotype, 1=incorrect because acronym was not resolved, 2=approximately correct, 3=fully correct.
    - o https://github.com/kuleshov/gwaskb/blob/master/notebooks/results/util/phenotype.mapping.gwascat.annotated.tsv

On the web interface (and perhaps elsewhere) variants are referred to as 'mutations'. The vast majority of variants examined in GWAS are common variants present in at least 5% of the population. It is completely inappropriate it refer to these as 'mutations'. Also note that there are many traits that are not 'diseases' in GWASkb.

We have corrected this.

• Typos in Supp methods (LD), PLINK should be capitalised

• Line 35 – "absent in all others", only two data sources were examined.

We have corrected these typos, and thank the reviewer for their attention to detail.

The authors should bear in mind some key differences between the GWAS Catalog and GWAS Central.

Note that not all GWAS Catalog and GWAS Central data is independently extracted. GWAS Central imports all GWAS Catalog data (GWAS Catalog does not import GWAS Central data). GWAS Central, I believe, does not do manual curation on all papers, in some cases the only data available is that taken from the GWAS Catalog.

GWAS Central also accepts user-submitted data, which has not necessarily appeared in the publication. Therefore there could be associations in GWAS Central which is not accessible to the authors' search algorithm.

REVIEWERS' COMMENTS:

Reviewer #1 (Remarks to the Author):

The authors have made a good effort to address the concerns raised by the reviewers, particularly reviewer 3, and the article now strikes a better balance of highlighting where a machine-compiled approach succeeds and fails. They now acknowledge that much of the meta-data around study design and power that manually curated resources such as GWAScatalog include, and that is required to quickly interpret an association, is missing from GWASkb. They have also included an extra meta-data table that will help users in getting to this data more quickly.

The striking claim in the original article of discovery of 1000's of new associations that had been "missed" by manual curation is now more extensively investigated, based on the feedback from reviewer 3. The new finding that most of these are deliberately excluded by the manual curators based on well-founded scientific guidelines is now properly described in the article. They correctly discuss the differences between a resource such as GWASkb that attempts to record nearly all possible GWAS associations and a high-quality, curated resource such as GWAScatalog and the pros and cons of both approaches.

In summary, the authors have taken all the feedback and now produced a well-balanced article and resource which will be of interest to the community.

Reviewer #2 – no comments to the authors -

Reviewer #3 (Remarks to the Author):

The authors have largely addressed most of my concerns. I am supportive of publication if they address my remaining points, these are mainly places where the authors did not fully address my initial concerns.

What is the title of the paper? It appears differently in different sections. I strongly favor ‘Machine-compiled’ over ‘Machine-curated’, curate implies the application of expert knowledge.

Major point 1. Improve internal referencing and accessibility of data.

Although the authors have made much improvements in providing their data, as I requested in my initial review, it is still extremely difficult and frustrating to find it and identify what is what. They have done a lot of work, but it is buried in a deep structure on github/supplementary files. File names are never referred to in the text. Explanatory notes are provided as README files.

If referring to data in the paper, please make it available in the supplemental text, give it a specific title (ideally a number) and refer to it specifically in the text (ie ‘on Github’ or ‘in supplement’ is NOT sufficient).

Provide a full table of contents (of all of github contents and supplemental text contents) in the supplemental text. This includes, but is not limited to, your ‘master list’ (https://github.com/kuleshov/gwaskb/blob/master/README.md).

I applaud the authors for inclusion of additional explanatory notes and figures in their response to my review, however these should also be made available in the supplementary file so that the full readership can benefit (e.g. the explanation of meta-data). In the specific case of the meta-data figure (where the extracted meta-data is discovery, replication) please add a second, less ideal, example (where the extracted meta-data is less immediately meaningful).

Major point 2. ‘Curator error’

In this revision the authors have re-assessed the associations found by GWASkb that are not present in the GWAS Catalog. Largely this analysis is much improved, and as suggested by my original review, find that most variants were excluded due to scientific reasons and comply with the GWAS Catalog extraction guidelines. The authors’ discussion of the scientific merits of excluding these variants is warranted and encouraged.

The authors describe ‘3 variants omitted due to what appears to be curator error’. However, I have examined these variants and found that they were all deliberately excluded as per the GWAS Catalog extraction guidelines (https://www.ebi.ac.uk/gwas/docs/methods/criteria). I have no doubt that there are curator errors in the GWAS Catalog, but none have been found in this analysis so this needs to be corrected.

These three variants (from the 100 SNP analysis) are:

PMID 19668339, rs429358

Comment: This association comes from an analysis focused on the APOE region (authors call it 'case-control analysis'), ie it is not a genome wide analysis and therefore not eligible for extraction for the GWAS Catalog.

Extraction guideline: “Studies and associations are eligible for inclusion in the GWAS Catalog if they meet the following criteria: * Include a primary GWAS analysis, defined as array-based genotyping and analysis of 100,000+ pre-QC SNPs selected to tag variation across the genome and without regard to gene content.”

PMID 24023777, rs6570507

Comment: This association was not replicated in the full cohort (not attempted for replication) ie deliberately excluded per extraction guidelines. The study design here is GWAS in Japanese, replication 1 in Japanese, replication 2 in Chinese. SNPs were extracted from T2 (GWAS + rep1 + rep2), this SNP was in T1 (GWAS + rep1 only).

PMID 22368281, rs6857

Comment: I specifically addressed this association in my first review, as it is highlighted by the authors in the text. I said “the final example association (rs6857) is reported by the authors as a ‘new’ association with Alzheimer’s and LDL cholesterol. … This association was not included in GWAS Catalog as it was not replicated.” Now adding: this replication is only present in the text, pg 1007, section 'replication of the LPA SNP'. rs6857 was not attempted for replication so not extracted.

Two of these variants, and comments in the text, suggest that the authors don’t fully understand that the GWAS Catalog extraction criteria regarding replication stage. If a replication stage is performed only significant associations from the combined are extracted. This means that a discovery association will be excluded if it is not attempted for replication. If no replication is performed, discovery data is extracted.

(The exception to this rule is if the broad ancestral categories of the discovery and replication cohorts are different, but that is not relevant in the cases the authors encounter.)

The authors also describe 7 new variants that were ‘found in a previous study and in the discovery stage of the current study, but not in its meta-analysis stage.’ Catalog guidelines state these should be extracted, but were not. The authors interpret this as ‘a small curator error’. I can clarify that the Catalog does not run any analysis to determine if an association is previously ‘known’ or not. Extraction is performed based on the publication only, so an association is classed as ‘known’ only if the author states in the paper it is known. I haven’t checked these particular variants, but it is likely that they had been previously found but that was not explicitly stated in the publication.

Major point 3. Advantages of human curation

While the authors have scaled back their claims regarding the superiority of machines over human curators and still advocate machine and human working together as the ideal scenario, I feel they do not adequately describe the advantages of human curation (see comments on lines 327, 353). I suggest adding description of advantages of manual curation (e.g. human curators are expert scientists who apply their expertise to interpret and distill often complex study design). One or two additional sentences on the richness of meta-data (study structure and design, rich and structured ancestry information, see PMID 29448949) curated by human curators would also be appropriate in the main text.

In light of no human curator error having been found, I suggest the authors reframe that discussion. Of course, human error is possible and I have no doubt that there are errors in the GWAS Catalog, so discussion of error is still valid. However it should be acknowledged that high quality manually curated resources such as the GWAS Catalog are trusted because a lot of effort goes in to ensuring the data are correct. In the case of the Catalog data are double curated (initial extraction by one curator which is fully reviewed by a second curator).

Minor points

Update GWAS Catalog citation to include the most recent publication (PMID 30445434). Is there are more up to date citation for GWAS Central (post 2013)?

Upon first reference to the Catalog please use its correct full name, the NHGRI-EBI GWAS Catalog.

Line 27 and throughout the paper - Variants not mutations. GWAS generally examines genetic variants that are present at at least 5% in the general population, therefore it is incorrect to refer to them as ‘mutations’.

Even if the variant is rare ‘mutation’ can be a problematic term. See HGVS guidelines ‘Mutation and polymorphism (In some disciplines the term "mutation" is used to indicate "a change" while in other disciplines it is used to indicate "a disease-causing change". Similarly, the term "polymorphism" is used both to indicate "a non disease-causing change" or "a change found at a frequency of 1% or higher in the population". To prevent this confusion we do not use the terms mutation and polymorphism (including SNP or Single Nucleotide Polymorphism) but use neutral terms like "sequence variant", "alteration" and "allelic variant". The Vol.19(1) issue of Human Mutation (2002) contains several contributions discussing these issues as well as the fact that the term "mutation" has developed a negative connotation (see Cotton RGH - p.2, Condit CM et al. - p.69 and Marshall JH - p.76). Therefore, current guidelines of authorative organisations now also recommend to use the neutral term "variant" only (e.g. Richards 2015, Genet.Med. 17:405-424).)

Line 19 vs Line 42 -basic result (recall percent) inconsistent between abstract and text

Line 62 – I understood associations are extracted from tables only?

Lines 140-145 – Clarification: The GWAS Catalog extracts combined (discovery and replication) if it is available, discovery data is extracted if no replication is attempted.

Line 156 –‘GWAS Central accepts all associations’ – is this the case? Do they accept non-genome-wide replication associations and present them on equal footing with discovery data (as GWASkb does)?

Line 163 -164 – I don’t understand this sentence.

Line 182 – please refer specifically to a file/location. I eventually found this file which does not contain column headers, please add and explain 0-3 ranking. How does this ranking correspond with ‘exact/approximate’ as described in text?

Line 194 -196 – this sentence is unclear. Suggest replacing ‘their’ with ‘the GWASkb phenotype was incorrect’ or similar. And replace ‘in GWAS Central/Catalog’ with ‘for GWAS Central/Catalog’.

Lines 212 -222 – the numbers here are slightly different to those provided in the supplement for the same analysis.

Discussion

Line 327 – the title (the importance of curation) does not reflect the content of the paragraph. The importance of curation is not actually discussed. Please amend.

Line 353 – the authors state that humans have many advantages over machines, but they do not describe any advantages. Please describe some advantages (e.g. human curators are expert scientists who apply their expertise to interpret and distill often complex study design)

Website:

The ‘source’ column is confusing, it suggests you are importing data from GWAS Catalog/central. Please clarify. OR/beta are sometimes present.. is this direct import data? If not OR/beta should be addressed in this paper.

REVIEWERS' COMMENTS:

Reviewer #1 (Remarks to the Author):

The authors have made a good effort to address the concerns raised by the reviewers, particularly reviewer 3, and the article now strikes a better balance of highlighting where a machine-compiled approach succeeds and fails. They now acknowledge that much of the meta-data around study design and power that manually curated resources such as GWAScatalog include, and that is required to quickly interpret an association, is missing from GWASkb. They have also included an extra meta-data table that will help users in getting to this data more quickly.

The striking claim in the original article of discovery of 1000's of new associations that had been "missed" by manual curation is now more extensively investigated, based on the feedback from reviewer 3. The new finding that most of these are deliberately excluded by the manual curators based on well-founded scientific guidelines is now properly described in the article. They correctly discuss the differences between a resource such as GWASkb that attempts to record nearly all possible GWAS associations and a high-quality, curated resource such as GWAScatalog and the pros and cons of both approaches.

In summary, the authors have taken all the feedback and now produced a well-balanced article and resource which will be of interest to the community.

Reviewer #2 – no comments to the authors -

Reviewer #3 (Remarks to the Author):

The authors have largely addressed most of my concerns. I am supportive of publication if they address my remaining points, these are mainly places where the authors did not fully address my initial concerns.

What is the title of the paper? It appears differently in different sections. I strongly favor ‘Machine-compiled’ over ‘Machine-curated’, curate implies the application of expert knowledge.

We have moved to machine-curated everywhere.

[BH] Major point 1. Improve internal referencing and accessibility of data.
Although the authors have made much improvements in providing their data, as I requested in my initial review, it is still extremely difficult and frustrating to find it and identify what is what. They have done a lot of work, but it is buried in a deep structure on github/supplementary files. File names are never referred to in the text. Explanatory notes are provided as README files. If referring to data in the paper, please make it available in the supplemental text, give it a specific title (ideally a number) and refer to it specifically in the text (ie ‘on Github’ or ‘in supplement’ is NOT sufficient). Provide a full table of contents (of all of github contents and supplemental text contents) in the supplemental text. This includes, but is not limited to, your ‘master list’ (https://github.com/kuleshov/gwaskb/blob/master/README.md).

We have updated our paper to now refer to an explicit numbered item in the Supplementary Material wherever data is referenced, included relevant code directly in the Supplementary Material document wherever feasible, and added the content of our supplementary code,

notebooks, etc. to our table of contents in the Supplemantary Material so that a reader can see all that is available from that single document.

I applaud the authors for inclusion of additional explanatory notes and figures in their response to my review, however these should also be made available in the supplementary file so that the full readership can benefit (e.g. the explanation of meta-data).

As suggested, we have added additional explanatory notes and figures from the response to reviewers to the Supplementary Materials. These include a more thorough description and example of meta-data extraction from tables (Supplementary Material 11), a screenshot of the web interface (Supplementary Material 12), and the error analysis of 100 extracted relations not present in GWAS Catalog (included in the Methods section of the main paper).

In the specific case of the meta-data figure (where the extracted meta-data is discovery, replication) please add a second, less ideal, example (where the extracted meta-data is less immediately meaningful).

We have added to the supplementary material (following the section that is referenced by the reviewer) a paragraph describing the three most common failure modes of the above approach of extracting data from the table columns.

Major point 2. 'Curator error'
In this revision the authors have re-assessed the associations found by GWASkb that are not present in the GWAS Catalog. Largely this analysis is much improved, and as suggested by my original review, find that most variants were excluded due to scientific reasons and comply with the GWAS Catalog extraction guidelines. The authors' discussion of the scientific merits of excluding these variants is warranted and encouraged.

The authors describe '3 variants omitted due to what appears to be curator error'. However, I have examined these variants and found that they were all deliberately excluded as per the GWAS Catalog extraction guidelines (https://www.ebi.ac.uk/gwas/docs/methods/criteria). I have no doubt that there are curator errors in the GWAS Catalog, but none have been found in this analysis so this needs to be corrected.

These three variants (from the 100 SNP analysis) are:

PMID 19668339, rs429358
Comment: This association comes from an analysis focused on the APOE region (authors call it 'case-control analysis'), ie it is not a genome wide analysis and therefore not eligible for extraction for the GWAS Catalog.
Extraction guideline: "Studies and associations are eligible for inclusion in the GWAS Catalog if they meet the following criteria: * Include a primary GWAS analysis, defined as array-based genotyping and analysis of 100,000+ pre-QC SNPs selected to tag variation across the genome and without regard to gene content."

PMID 24023777, rs6570507
Comment: This association was not replicated in the full cohort (not attempted for replication) ie deliberately excluded per extraction guidelines. The study design here is GWAS in Japanese, replication 1 in Japanese, replication 2 in Chinese. SNPs were extracted from T2 (GWAS + rep1 + rep2), this SNP was in T1 (GWAS + rep1 only).

PMID 22368281, rs6857
Comment: I specifically addressed this association in my first review, as it is highlighted by the authors in the text. I said “the final example association (rs6857) is reported by the authors as a ‘new’ association with Alzheimer’s and LDL cholesterol. … This association was not included in GWAS Catalog as it was not replicated.” Now adding: this replication is only present in the text, pg 1007, section 'replication of the LPA SNP'. rs6857 was not attempted for replication so not extracted.

Two of these variants, and comments in the text, suggest that the authors don’t fully understand that the GWAS Catalog extraction criteria regarding replication stage. If a replication stage is performed only significant associations from the combined are extracted. This means that a discovery association will be excluded if it is not attempted for replication. If no replication is performed, discovery data is extracted.
(The exception to this rule is if the broad ancestral categories of the discovery and replication cohorts are different, but that is not relevant in the cases the authors encounter.)

The authors also describe 7 new variants that were ‘found in a previous study and in the discovery stage of the current study, but not in its meta-analysis stage.’ Catalog guidelines state these should be extracted, but were not. The authors interpret this as ‘a small curator error’. I can clarify that the Catalog does not run any analysis to determine if an association is previously ‘known’ or not. Extraction is performed based on the publication only, so an association is classed as ‘known’ only if the author states in the paper it is known. I haven’t checked these particular variants, but it is likely that they had been previously found but that was not explicitly stated in the publication.

We thank the reviewer for catching these errors. Our inclusion of these variants was due to a misunderstanding of the methodology used in the paper (which was not as clearly described as in other cases). We have now corrected the references to these 3 variants throughout the paper.

Major point 3. Advantages of human curation
While the authors have scaled back their claims regarding the superiority of machines over human curators and still advocate machine and human working together as the ideal scenario, I feel they do not adequately describe the advantages of human curation (see comments on lines 327, 353). I suggest adding description of advantages of manual curation (e.g. human curators are expert scientists who apply their expertise to interpret and distill often complex study design). One or two additional sentences on the richness of meta-data (study structure and design, rich and structured ancestry information, see PMID 29448949) curated by human curators would also be appropriate in the main text.

In light of no human curator error having been found, I suggest the authors reframe that discussion. Of course, human error is possible and I have no doubt that there are errors in the GWAS Catalog, so discussion of error is still valid. However it should be acknowledged that high quality manually curated resources such as the GWAS Catalog are trusted because a lot of effort goes in to ensuring the data are correct. In the case of the Catalog data are double curated (initial extraction by one curator which is fully reviewed by a second curator).

We have updated the text to remove the claim of out-performing humans. We have also added additional sections to the Discussion that adopt all the recommendaitons made by the reviewer, and in particular that describe the strenths of human curation.

Minor points

Upon first reference to the Catalog please use its correct full name, the NHGRI-EBI GWAS Catalog.

Done.

Line 27 and throughout the paper - Variants not mutations. GWAS generally examines genetic variants that are present at at least 5% in the general population, therefore it is incorrect to refer to them as ‘mutations’.

Even if the variant is rare ‘mutation’ can be a problematic term. See HGVS guidelines ‘Mutation and polymorphism (In some disciplines the term "mutation" is used to indicate "a change" while in other disciplines it is used to indicate "a disease-causing change". Similarly, the term "polymorphism" is used both to indicate "a non disease-causing change" or "a change found at a frequency of 1% or higher in the population". To prevent this confusion we do not use the terms mutation and polymorphism (including SNP or Single Nucleotide Polymorphism) but use neutral terms like "sequence variant", "alteration" and "allelic variant". The Vol.19(1) issue of Human Mutation (2002) contains several contributions discussing these issues as well as the fact that the term "mutation" has developed a negative connotation (see Cotton RGH - p.2, Condit CM et al. - p.69 and Marshall JH - p.76). Therefore, current guidelines of authorative organisations now also recommend to use the neutral
term "variant" only (e.g. Richards 2015, Genet.Med. 17:405-424).)

We have changed all occurences of mutation to variant.

Line 19 vs Line 42 -basic result (recall percent) inconsistent between abstract and text
We updated these to be consistent.

Line 62 – I understood associations are extracted from tables only?

Yes, and we clarified this.

Lines 140-145 – Clarification: The GWAS Catalog extracts combined (discovery and replication) if it is available, discovery data is extracted if no replication is attempted.

We added the clarificaiton to the text.

Line 156 –‘GWAS Central accepts all associations’ – is this the case? Do they accept non-genome-wide replication associations and present them on equal footing with discovery data (as GWASkb does)?

They do not thoroughly check what the users submit. We clarified the text.

Line 163 -164 – I don’t understand this sentence.

We rephrased it more clearly.

Line 182 – please refer specifically to a file/location. I eventually found this file which does not contain column headers, please add and explain 0-3 ranking. How does this ranking correspond with ‘exact/approximate’ as described in text?

The ranking corresponds to 0=incorrect, 1=correct but with wrong phenotype, 2=correct but imprecise phenotype, 3=fully correct. We clarified this.

Line 194 -196 – this sentence is unclear. Suggest replacing ‘their’ with ‘the GWASkb phenotype was incorrect’ or similar. And replace ‘in GWAS Central/Catalog’ with ‘for GWAS Central/Catalog’.

Done.

Lines 212 -222 – the numbers here are slightly different to those provided in the supplement for the same analysis.

Unfortunately, we cannot see the line numbers the reviewer is referring to and were unable to identify the discrepancy. With a little more context, we would be happy to take a closer look and correct any differences.

Discussion
Line 327 – the title (the importance of curation) does not reflect the content of the paragraph. The importance of curation is not actually discussed. Please amend.

Amended.

Line 353 – the authors state that humans have many advantages over machines, but they do not describe any advantages. Please describe some advantages (e.g. human curators are expert scientists who apply their expertise to interpret and distill often complex study design)

We added a paragraph.

Website:
The ‘source’ column is confusing, it suggests you are importing data from GWAS Catalog/central. Please clarify. OR/beta are sometimes present.. is this direct import data? If not OR/beta should be addressed in this paper.

The reviewer is correct; we are indeed importing data and OR/beta from source wherever this information is available for the entity that is being reported on.
